# ERF109 of trifoliate orange (*Poncirus trifoliata* (L.) Raf.) contributes to cold tolerance by directly regulating expression of *Prx1* involved in antioxidative process

**DOI:** 10.1111/pbi.13056

**Published:** 2019-01-04

**Authors:** Min Wang, Wenshan Dai, Juan Du, Ruhong Ming, Bachar Dahro, Ji‐Hong Liu

**Affiliations:** ^1^ Key Laboratory of Horticultural Plant Biology College of Horticulture and Forestry Sciences Huazhong Agricultural University Wuhan China

**Keywords:** ethylene‐responsive factors, *Poncirus trifoliata*, cold tolerance, citrus, transcriptional regulation, peroxidase, reactive oxygen species

## Abstract

Ethylene‐responsive factors (ERFs) have been revealed to play essential roles in a variety of physiological and biological processes in higher plants. However, functions and regulatory pathways of most ERFs in cold stress remain largely unclear. Here, we identified *PtrERF109* of trifoliate orange (*Poncirus trifoliata* (L.) Raf.) and deciphered its role in cold tolerance. *PtrERF109* was drastically up‐regulated by cold, ethylene and dehydration, but repressed by salt. PtrERF109 was localized in the nucleus and displayed transcriptional activity, and the C terminus is required for the activation. Overexpression of *PtrERF109* conferred enhanced cold tolerance in transgenic tobacco and lemon plants, whereas VIGS (virus‐induced gene silencing)‐mediated suppression of *PtrERF109* in trifoliate orange led to increased cold susceptibility. *PtrERF109* overexpression caused extensive transcriptional reprogramming of several suites of stress‐responsive genes. *Prx1* encoding class III peroxidase (POD) was one of the antioxidant genes exhibiting the greatest induction. PtrERF109 was shown to directly bind to the promoter of *PtrPrx1* (trifoliate orange *Prx1* homologue) and positively activated its expression. In addition, the *PtrERF109*‐overexpressing plants exhibited significantly higher POD activity and accumulated dramatically less H_2_O_2_ and were more tolerant to oxidative stress, whereas the VIGS plants exhibited opposite trends, in comparison with wild type. Taken together, these results indicate that PtrERF109 as a positive regulator contributes to imparting cold tolerance by, at least partly, directly regulating the POD‐encoding gene to maintain a robust antioxidant capacity for effectively scavenging the reactive oxygen species. Our findings gain insight into better understanding of transcriptional regulation of antioxidant genes in response to cold stress.

## Introduction

Citrus is one of the most important fruit crops throughout the world. According to the statistics of FAO, the worldwide citrus producing area in 2016 was 9.73 million hectares with a yield of more than 146.15 million tons, most of which were produced in China, India, Nigeria, Brazil, Mexico, USA, Spain, Egypt, Italy and Argentina. Thus, a sustainable and stable citrus industry is required for the producing regions. However, citrus production is constantly challenged by a variety of unfavourable environmental cues, including biotic and abiotic stresses. Cold is one of the major environmental factors that adversely influences the survival, growth and development of plants and greatly reduces crop productivity and quality. Being of subtropical origin, citrus is vulnerable to the cold stress; therefore, improving cold tolerance has been long regarded as an important target for breeding program. Trifoliate orange (*Poncirus trifoliata* (L.) Raf.) is closely related to citrus and extremely cold hardy (Sahin‐Cevik, 2013). Since trifoliate orange is cross‐compatible with citrus, it is naturally conceivable to cross them in order to produce cold‐tolerant progenies. Unfortunately, this traditional means is substantially impeded due to unique reproductive characteristics of the majority of citrus cultivars, including polyembryony, long juvenile period, high degree of heterozygosity and male/female sterility (Zhang *et al*., 2018). As an alternative strategy, genetic engineering has been increasingly demonstrated to act as an effective and efficient approach to generate transgenic plants with improved stress tolerance. To this end, it is imperative to identify potential genes that play critical roles in cold responses.

Plants have evolved a set of versatile adaptive mechanisms, causing an array of physiological, biochemical and metabolic changes, to cope with harsh environment (Zhu, 2016). Exposure to cold stress has been shown to trigger extensive reorganization of the global transcriptome (Thomashow, 2010), in which transcription factors have been well known as master switches for transducing external environmental stimuli into cellular response (Liu *et al*., 2014). As a single transcription factor can simultaneously regulate an array of target genes the TFs are regarded as proper candidates to genetically manipulate stress tolerance. So far, many TFs from different families have been well characterized to impart abiotic stress tolerance (Liu *et al*., 2014). Over the last two decades, tremendous achievements have been made to decipher the transcriptional network implicated in the cold stress response. The best understood regulatory cascade consists of three C‐repeat (CRT) binding factors (CBFs) and their downstream *COR* (cold‐regulated) genes, collectively called as CBF regulon. A number of studies demonstrate that the CBFs play pivotal roles in rendering plants able to tolerate the cold stress by interacting with CRT/DRE *cis*‐acting elements in the promoter of the *COR* genes (Liu *et al*., 2014). As plants contain a large spectrum of transcription factors in their genomes it is conceivable that plants may not completely depend upon the CBF regulatory module to control the *COR* genes for combating the cold stress. This extrapolation is supported by several lines of evidence based on the genome‐wide transcriptome analyses. For example, Fowler and Thomashow (2002) reported that nearly 12% of the *Arabidopsis thaliana COR* genes were controlled by CBFs. Recently, Park *et al*. (2015) demonstrated that only 172 out of 2637 *COR* genes (6.5% of the total) were designated as the CBF regulon. These findings suggest that only a small part of the *COR* genes is regulated by the well‐characterized CBFs and that other transcription factors participate in the transcriptional regulation of the *COR* genes. As a matter of fact, a number of transcription factors other than the CBFs have been shown to play essential roles in orchestration of the cold signalling, including those from the families of MYB (Xie *et al*., 2018), homeobox protein (Zhu *et al*., 2004), WRKY (Zou *et al*., 2010), basic helix‐loop‐helix (bHLH) proteins (Geng and Liu, 2018; Huang *et al*., 2013), and NAC (Hu *et al*., 2008a). These transcription factors modulate the cold tolerance through either CBF‐dependent or CBF‐independent manners, or both in some cases. However, it is reasonable to assume that other transcription factors might be also implicated in the cold stress signalling given the presence of large number of such regulators in the plant genome.

The APETALA2/ethylene‐responsive factors (AP2/ERFs) are one of the largest groups of plant‐specific transcription factors that are characterized by presence of one or two AP2/ERF domains composed of approximately 60 conserved amino acids. The AP2/ERF proteins contain four major subfamilies: DREB, ERF, AP2 and RAV, each harbouring unique conserved motifs (Nakano *et al*., 2006). A number of studies reveal that the ERF subfamily members in various plant species, acting as either transcriptional activators or repressors, play a pivotal role in regulation of a range of physiological and biological processes, including internode elongation (Zhou *et al*., 2016), root growth (Jung *et al*., 2017), trichome formation (Sun *et al*., 2017), fruit ripening (Yin *et al*., 2016), hormone signal transduction (Rashotte *et al*., 2006), secondary metabolism and biotic stress response (Zeng *et al*., 2015; Zhu *et al*., 2014). Accumulating evidence demonstrate that ERFs are also implicated in response to abiotic stresses, including submergence or hypoxia (Xu *et al*., 2006), heavy metal (Lin *et al*., 2017), drought (Jung *et al*., 2017) and high salinity (Yao *et al*., 2016). ERFs have been also demonstrated to be responsive to cold stress. For example, nine out of 95 TFs that were up‐regulated by cold in *Arabidopsis thaliana* were categorized into the ERF subfamily (Lee *et al*., 2005). In a recent study, Bolt *et al*. (2017) showed that *ERF105* of *Arabidopsis thaliana* was required for freezing tolerance and cold acclimation. Nevertheless, it is worth mentioning that there is an enormous paucity of knowledge concerning the function and the underlying regulatory network of the ERFs in cold tolerance although some cold‐inducible ERF transcription factors have been reported in various plants (Sharma *et al*., 2010; Sharoni *et al*., 2011).

We previously carried out a time‐course transcriptome analysis using cold‐treated *Poncirus trifoliata* and identified 60 cold‐inducible TFs, including 12 ERF genes, among which Unigene20109 encodes an ERF protein and exhibited dramatic up‐regulation during cold treatment (Wang *et al*., 2015). However, the function of this gene, denoted as *PtrERF109*, remains unclear. In this study, we investigated the role of *PtrERF109* in cold tolerance by overexpression and VIGS (virus‐induced gene silencing). We carried out RNA sequencing (RNA‐Seq) in order to identify genes that could be potentially regulated by PtrERF109. Interestingly, a peroxidase (POD)‐encoding gene *PtrPrx1* was confirmed to be directly regulated by PtrERF109. Consistently, overexpression of *PtrERF109* increased POD activity, concurrent with reduced reactive oxygen species (ROS) accumulation and enhanced oxidative stress tolerance, whereas silencing of *PtrERF109* had the opposite impacts. Collectively, our findings demonstrate that PtrERF109 plays a positive role in cold tolerance, which is ascribed to, at least in part, modulation of ROS homoeostasis by directly regulating the *Prx1* gene.

## Results

### Cloning and bioinformatics analysis of *PtrERF109*

**Figure 1:**
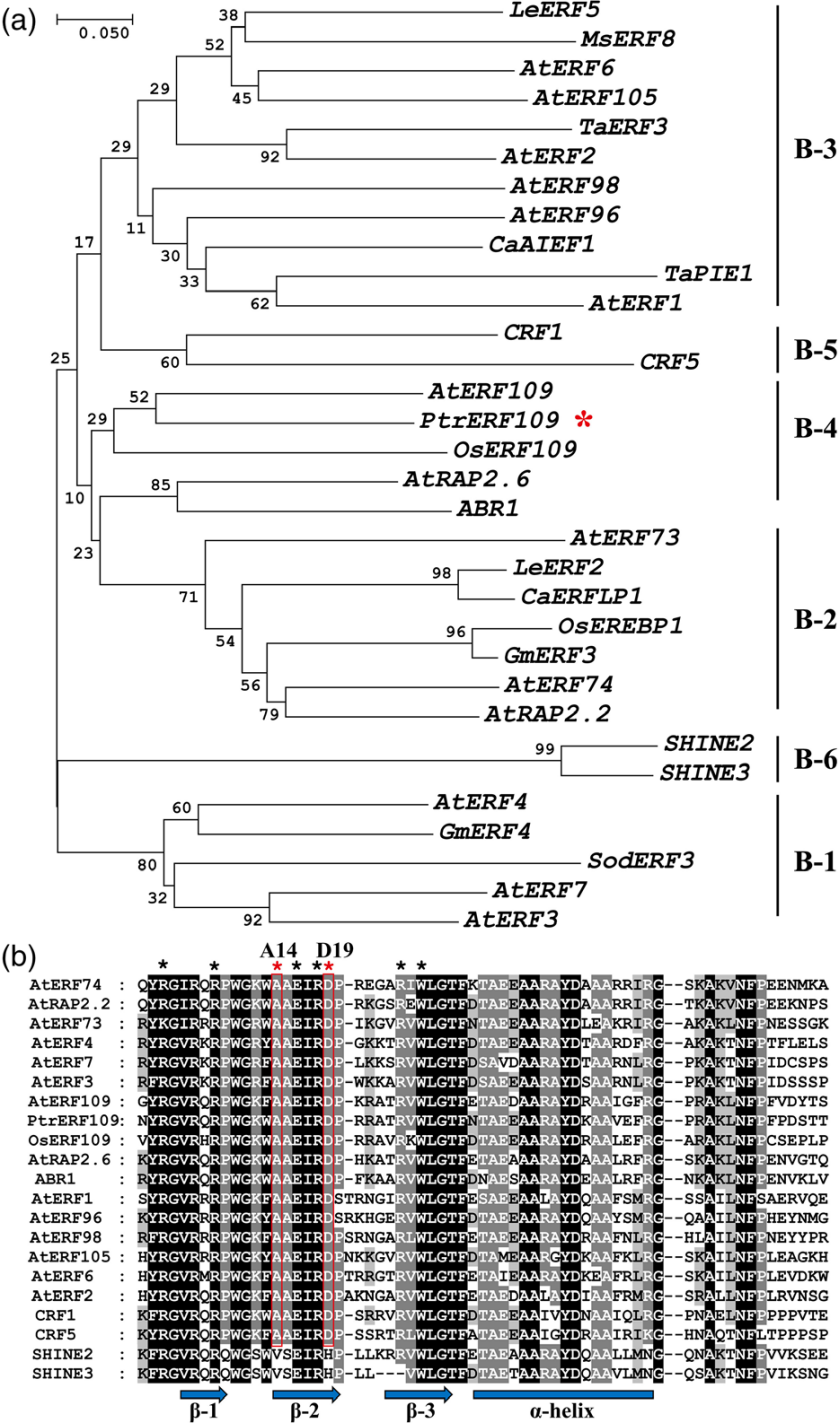
Phylogenetic analysis and sequence alignments of PtrERF109 and ERFs from other plants. (a) A neighbour‐joining phylogenetic tree constructed based on amino acids of PtrERF109 (marked with an asterisk) and ERFs of *Arabidopsis thaliana* (prefixed with At), *Oryza sativa* L. (Os), *Lycopersicon esculentum* (Le), *Glycine max* (Gm), *Medicago sativa* (Ms), *Triticum aestivum* (Ta), *Saccharum officinarum* (So), and *Capsicum annuum* (Ca). B‐1 to B‐6 represent six subgroups of ERF subfamily members. (b) Multiple alignments of the AP2 domains of PtrERF109 and ERFs from other plants. Identical and conserved amino acid residues are highlighted with black and grey shade, respectively. Black asterisks indicate the residues for DNA binding, while the red ones indicate the two characteristic residues for ERF subfamily. The arrows and bar indicate β‐sheets and α‐helix regions, respectively.

The full‐length *PtrERF109* was amplified by PCR based on the sequence of Unigene20109, which was identified previously (Wang *et al*., 2015). The ORF (open reading frame) of *PtrERF109* is 927 bp, encoding a putative protein of 308 amino acids (aa) with a predicted molecular mass of 34.1 kDa and an isoelectric point of 8.29. The predicted protein contained a conserved 64‐aa AP2/ERF domain. A phylogenetic tree was constructed based on AP2/ERF domains of PtrERF109 and 21 ERF proteins from other plant species (Figure 1a), in which *PtrERF109* was most closely related to *ERF109* of *A. thaliana* (AT4G34410) and rice (XP_015649367.1). This is why the transcription factor was designated as *PtrERF109*. Of note, the residues at the 14th and 19th sites in the AP2/ERF domain of PtrERF109 were alanine and aspartic acid, respectively (Figure 1b), which are characteristic feature for the ERF subfamily, implying that PtrERF109 was categorized in the ERF subfamily. In addition, we found that PtrERF109 shares high sequence similarity of the AP2 domains with its homologues from other citrus species and its relatives, including *Citrus sinensis*,* Citrus clementina*,* Citrus limon, Citrus medica*,* Citrus grandis*,* Citrus ichangensis*,* Citrus reticulata*,* Fortunella crassifolia* and *Atlantia buxifolia*, in which only two amino acids were different (Figure S1). PtrERF109 sequence has been deposited in GenBank with the accession number MH779873.

### Expression patterns of *PtrERF109*

**Figure 2:**
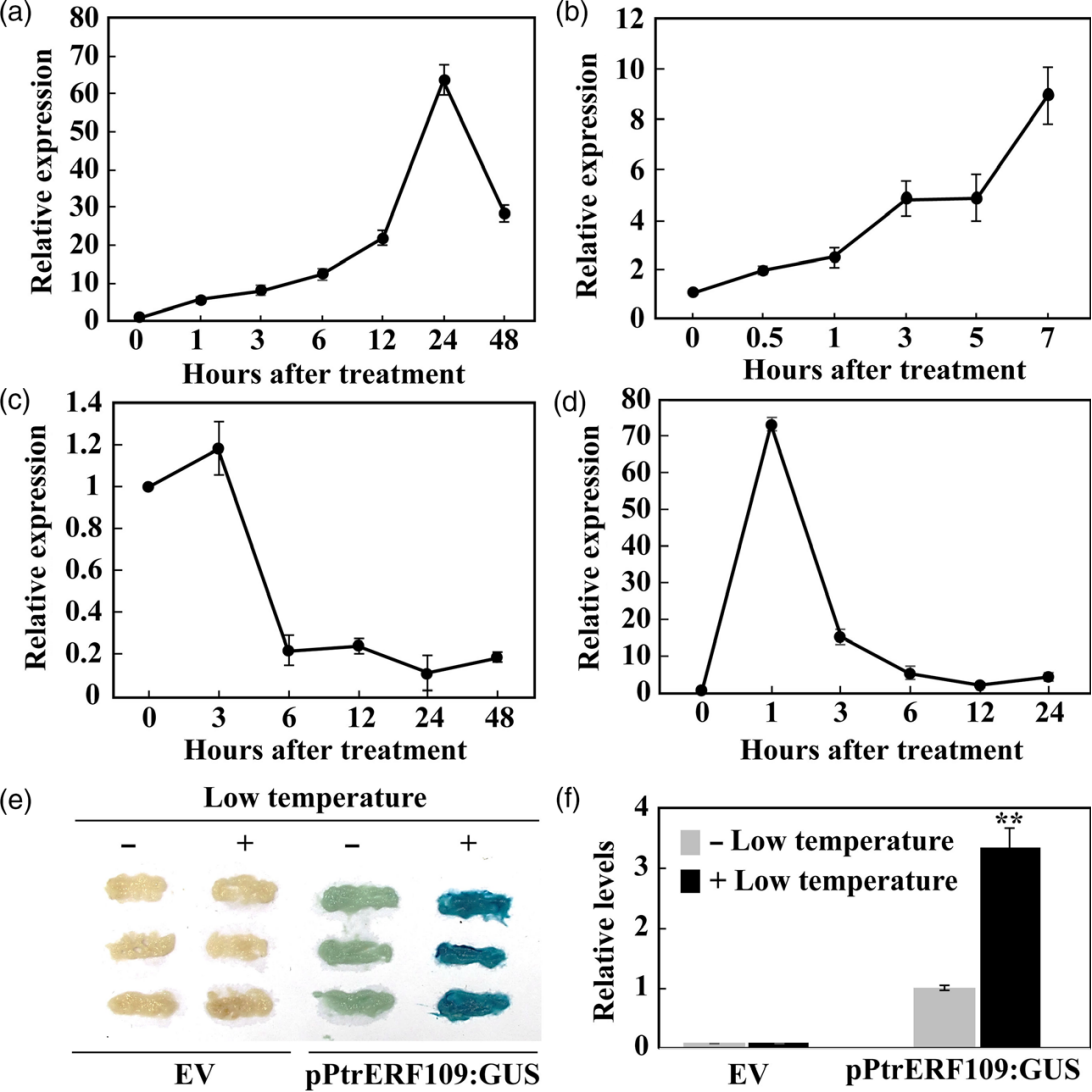
Expression profiles of *PtrERF109* under various treatments. (a–d) Expression patterns of *PtrERF109* in response to cold (a), dehydration (b), salt (c), and ethephon (d), as analysed by quantitative real‐time PCR analysis. *Actin* was used as an internal control. Error bars represent ± SE (*n* = 3). (e, f) GUS staining (e) and relative GUS intensity (f) of sweet orange callus transiently transformed with pPtrERF109:GUS or empty vector (EV) with (+) or without (−) cold treatment. Error bars represent ± SE (*n* = 3). Asterisks indicate significant difference before and after the cold treatment (**P < 0.01).

Expression profiles of *PtrERF109* in response to abiotic stresses and exogenous ethylene treatment was examined by qPCR. *PtrERF109* transcript levels were progressively enhanced by cold treatment, peaking at the 24th hour (induced by more than 60 folds), followed by a decrease to nearly 30 folds of the initial level (Figure 2a). Upon exposure to dehydration, *PtrERF109* mRNAs exhibited steady up‐regulation during a 7‐h period, with >8‐fold increase relative to the onset of treatment (Figure 2b). In the presence of salt stress, *PtrERF109* underwent negligible change in gene expression within 3 h, but displayed a sharp down‐regulation at 6 h and then maintained stable thereafter (Figure 2c). Treatment with ethephon for 1 h led to a quick and drastic elevation of *PtrERF109* mRNA abundance (by more than 70 folds), which exhibited continuous decrease from 3 h onwards (Figure 2d). It seems that *PtrERF109* was induced to the greatest degree by cold treatment. In order to confirm the cold‐induced up‐regulation of *PtrERF109*, a transient expression study was performed by expressing *GUS* reporter gene driven by *PtrERF109* promoter (pPtrERF109) in citrus callus. Histochemical staining showed that the GUS expression was detected in calluses transformed with pPtrERF109:GUS in the absence of cold (4 °C) treatment, whereas the intensity was significantly elevated when the callus was subjected to the cold conditions (Figure 2e, f), indicating that *PtrERF109* was truly responsive to cold.

We also compared expression levels of *PtrERF109* and *ClERF109*, an *ERF109* gene of lemon (*Citrus lemon*), which is a cold‐sensitive species, using seedlings exposed to low temperature treatment. Both *PtrERF109* and *ClERF109* were induced by cold within 24 h, followed by a decline at 72 h of treatment. However, it is noticeable that *PtrERF109* of trifoliate orange showed substantially higher levels than its counterpart of lemon during the cold treatment, in particular within the first 24 h (Figure S2).

### PtrERF109 is a nucleic protein with transcriptional activation activity

**Figure 3:**
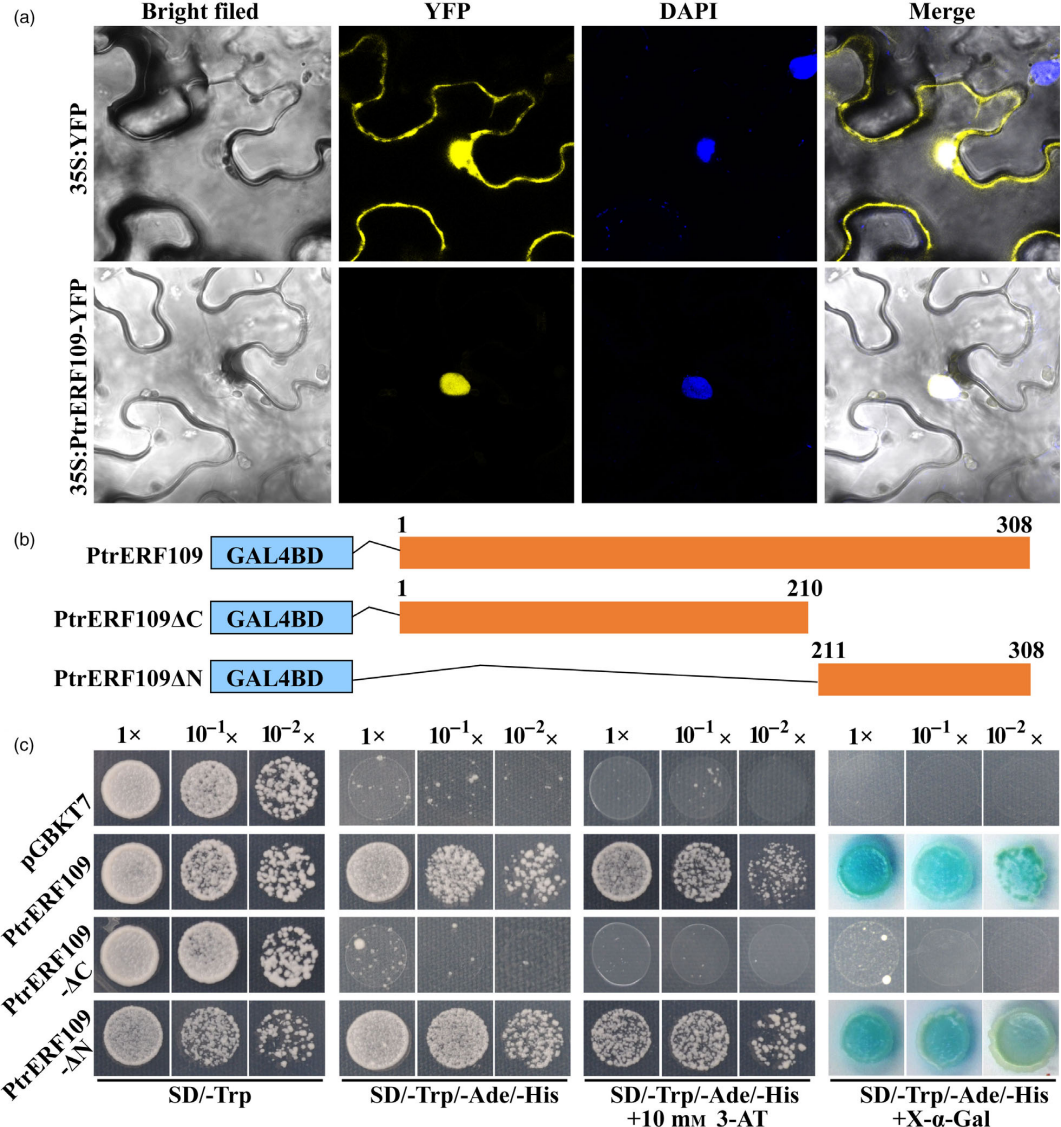
Subcellular localization and transcriptional activity assays of PtrERF109. (a) PtrERF109 is localized in the nucleus, based on visualization of yellow fluorescent protein (YFP) in tobacco (*Nicotiana benthamiana*) leaves transformed with a fusion construct (35S:PtrERF109‐YFP) or empty vector (35S:YFP). Microscopic images were taken under bright filed and fluorescence. DAPI was used to stain the nucleus. The overlapped images are shown on the right. (b) Schematic diagrams of constructs used for transcriptional activity assays. Full‐length or truncated (PtrERF109ΔC and PtrERF109ΔN indicate deletion of the C and N terminus, respectively) *PtrERF109* was introduced at the downstream of the GAL4 DBD in pGBKT7 vector. The numbers above the bars indicate the positions of amino acid residues. (c) Growth of yeast cells co‐transformed with different vectors on the selective medium added with or without X‐α‐Gal. Transformation of pGBKT7 vector was used as a negative control. 1×, 10^−1^× and 10^−2^× represent yeasts with original concentration (OD_600_ = 0.4), 10 and 100 folds of dilution, respectively.

To analyse the subcellular localization of PtrERF109, full‐length *PtrERF109* ORF without the stop codon was fused in‐frame to the 5′ end of YFP (yellow fluorescent protein) under the control of the CaMV (cauliflower mosaic virus) *35S* promoter. Microscopic observation showed that the YFP signal was evenly distributed in the cell when the control vector (*35S:YFP)* was transiently expressed in tobacco leaves. Nevertheless, the fusion protein PtrERF109‐YFP was exclusively expressed in the nucleus, indicating that PtrERF109 is localized in the nuclei (Figure 3a).

To determine whether PtrERF109 has transcriptional activation activity, either full‐length or truncated PtrERF109 was fused downstream to the GAL4‐binding domain (BD) in the pGBKT7 vector and transformed into the yeast (Figure 3b). All of the yeast cells showed normal growth on SD/‐Trp medium, whereas only the yeast cells transformed with both BD‐PtrERF109 and BD‐PtrERF109ΔN vectors survived when they were cultured on selective medium SD/‐Trp/‐Ade/‐His added with or without 10 mm 3‐AT. In addition, these two fusion proteins, but not the negative control and BD‐PtrERF109ΔC, were able to activate expression of the reporter gene *MEL1* (Figure 3c), indicating that PtrERF109 has transactivation activity and the transactivation domain was located in its C‐terminal end.

### Overexpression of *PtrERF109* confers enhanced cold tolerance

**Figure 4:**
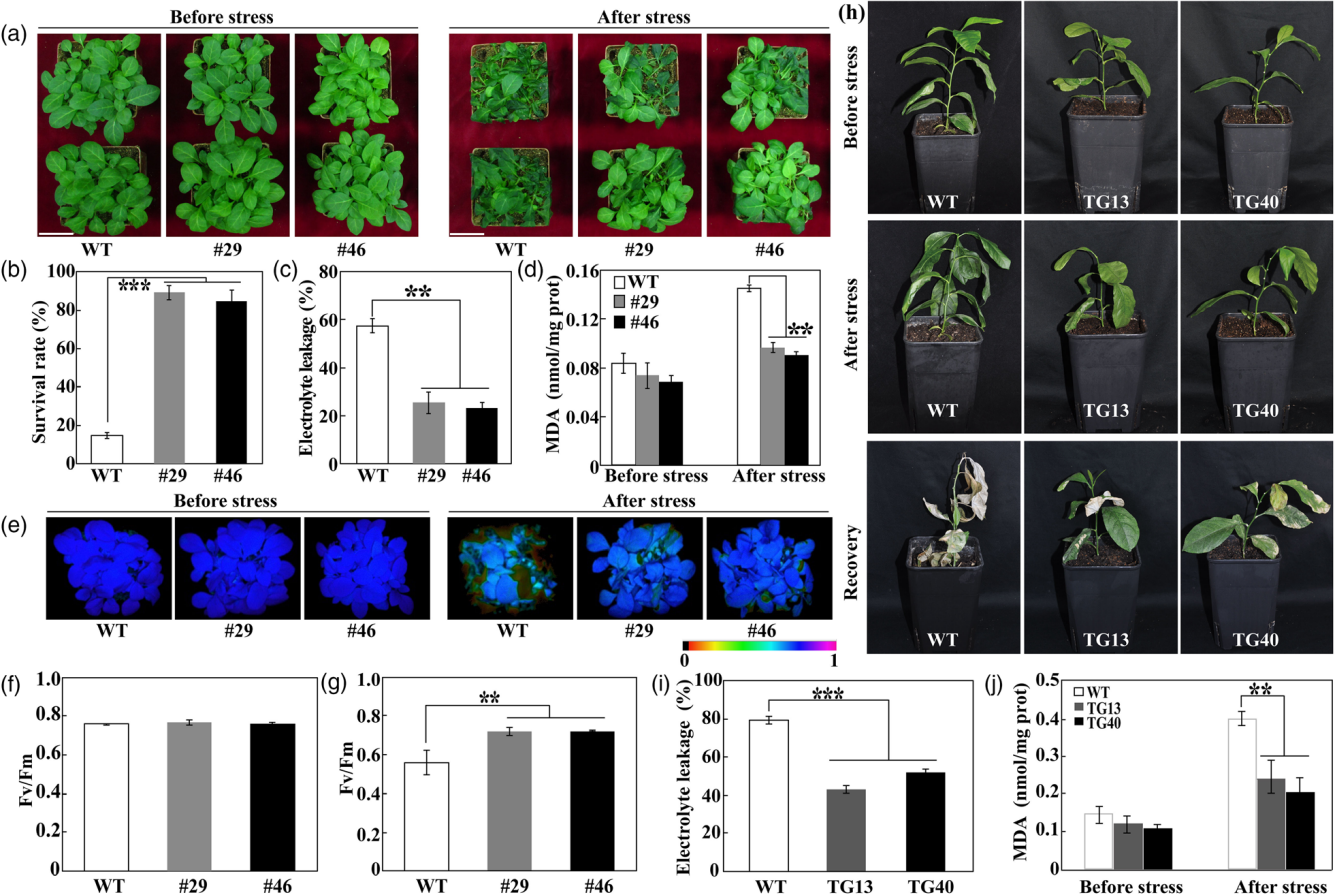
Overexpression of *PtrERF109* conferred enhanced cold tolerance in transgenic plants. (a) Phenotypes of tobacco transgenic lines (#29 and #46) and wild type (WT) before and after cold treatment. (b–d) Survival rate (b), electrolyte leakage (EL, c) and malondialdehyde (MDA) contents (d) of the WT and transgenic lines. (e–g) Chlorophyll fluorescence imaging (e) and *F*_v_/*F*_m_ ratios (f, g) of the WT and transgenic lines before and after cold treatment. (h) Phenotypes of lemon transgenic lines and wild type (WT) before and after cold treatment (8 h at −4 °C), followed by recovery for 14 day at ambient environment. (i, j) EL (i) and MDA contents (j) of transgenic lemon lines and WT. Error bars represent ± SE (*n* = 3). Asterisks indicate significant differences between transgenic lines and WT (**P < 0.01, ***P < 0.001).

The fact that *PtrERF109* was dramatically induced seems to suggest that *PtrERF109* may play a critical role in regulation of cold response. To verify this assumption, two transgenic tobacco lines overexpressing *PtrERF109*, designated as #29 and #46, were generated via *Agrobacterium tumefaciens*‐mediated transformation (Figure S3). Cold tolerance of the tobacco transgenic lines was assessed using 30‐day‐old plants grown in soil pots. Under normal conditions, there was no obvious morphological difference between the transgenic lines and the wild type (WT). Cold stress assays indicated that the transgenic lines were more resistant to freezing treatment than the WT. Morphologically, the WT suffered more severe plant injury in comparison with the transgenic lines (Figure 4a). Compared with 14.7% survival rate in the WT, 89.1% of #29 and 84.3% of #46 transgenic plants survived after a 12‐h freezing treatment at −2 °C followed by a 7‐day recovery at ambient environment (Figure 4b). Electrolyte leakage (EL) and malondialdehyde (MDA) were used to indicate cell injuries caused by the stresses. Both EL and MDA levels in the transgenic lines were significantly lower relative to the WT in the presence of cold treatment despite the comparable level of MDA between each other without stress treatment (Figure 4c, d), suggesting that cell injury was greater in the WT than in the transgenic lines. Maximum quantum yield of PSII (*F*_v_/*F*_m_ ratios), a valuable criterion for evaluation of photoinhibition in plants subjected to environmental stresses, was also examined using fluorescence imaging. Before cold stress the tested lines exhibited similar fluorescence imaging, which was impaired to greater degree in the WT compared with the transgenic plants under the cold stress (Figure 4e). In agreement with the imaging profile, *F*_v_/*F*_m_ ratio of WT was equivalent to that of the transgenic lines under normal condition, but was significantly lower in the presence of cold treatment (Figure 4f, g). These results demonstrate that overexpression of *PtrERF109* dramatically enhances cold tolerance in transgenic tobacco.

The role of *PtrERF109* in cold tolerance was then investigated by overexpressing *PtrERF109* in lemon (*C. limon*), a cold‐sensitive citrus species (Figure S4). Two lemon transgenic lines (TG13 and TG40), together with WT, were exposed to −4 °C for 8 h, followed by recovery for 14 day at 25 °C. Few of the lemon WT plants survived at the end of recovery, whereas most of the transgenic plants recovered their growth although leaf damage was also observed (Figure 4h). Consistent with the morphological observation, EL and MDA levels in the transgenic lines were prominently and significantly lower in comparison with those of the WT (Figure 4i, j). These results indicate that overexpression of *PtrERF109* drastically improved the cold tolerance of transgenic lemon.

### Silencing of *PtrERF109* in *Poncirus trifoliata* increases cold sensitivity

**Figure 5:**
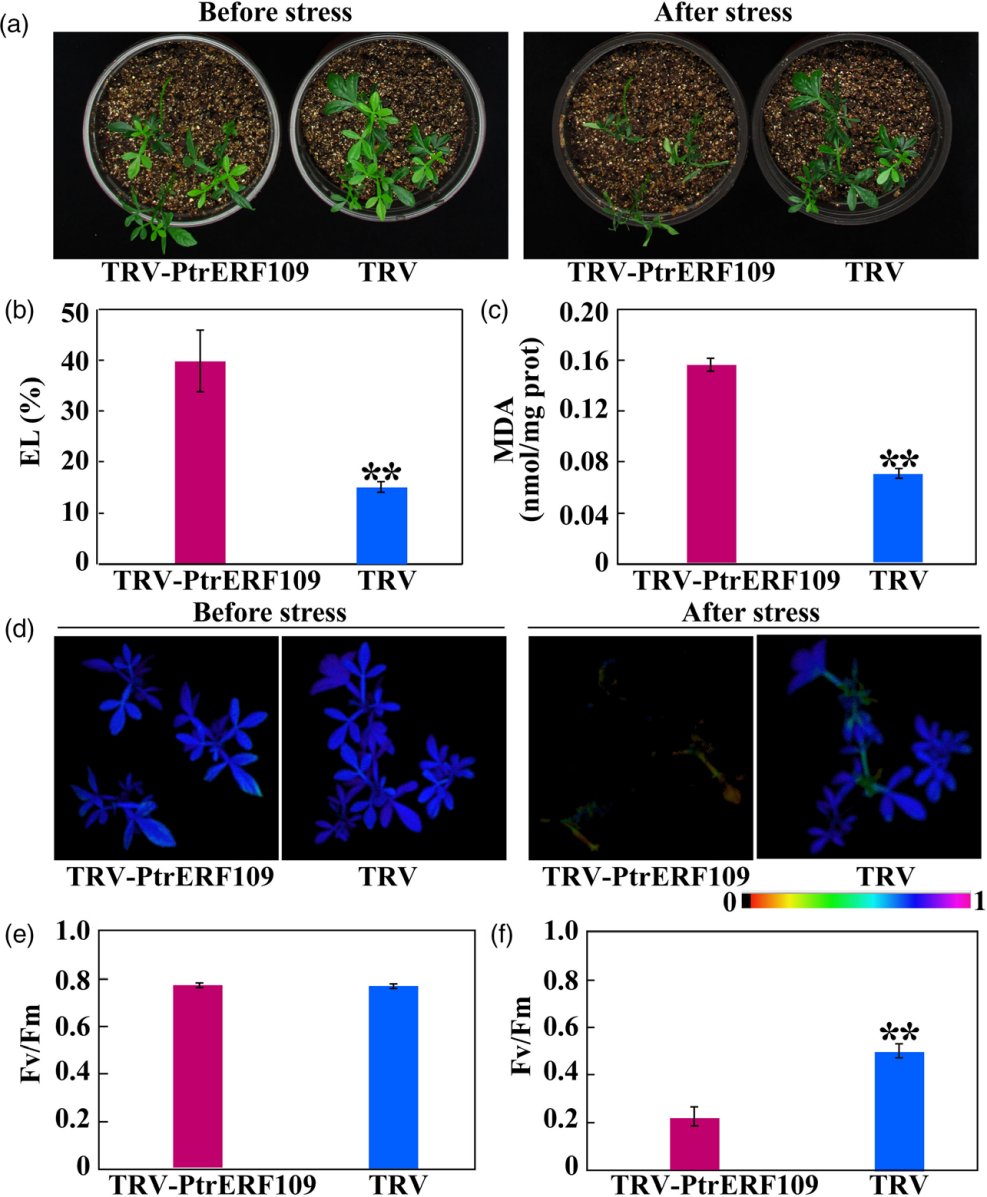
Silencing of *PtrERF109* causes enhanced cold sensitivity in trifoliate orange. (a) Phenotypes of VIGS plants (TRV‐PtrERF109) and control plants (TRV) before and after cold treatment (12 h at −2 °C). (b, c) EL (b) and MDA contents (c) of VIGS plants and TRV control after the cold treatment. (d‐f) Chlorophyll fluorescence imaging (d) and *F*_v_/*F*_m_ ratios (e, f) of VIGS plants and TRV control before and after the cold treatment. Error bars represent ± SE (*n* = 3). Asterisks indicate significant differences between VIGS (TRV‐PtrERF109) and TRV control plants (**P < 0.01).

To further elucidate the role of *PtrERF109* in cold tolerance, VIGS was used to knock down *PtrERF109* in trifoliate orange. Transcript abundance of *PtrERF109* in the positive VIGS plants, designated as TRV (tobacco rattle virus)‐PtrERF109, were repressed, ranging from 18% to 57%, compared to the control seedlings (designated as TRV) that were only infiltrated with empty vector (Figure S5). No conspicuous differences in plant morphology were observed between the VIGS and the TRV plants under normal growth conditions. In contrast, when they were exposed to freezing temperature (−2 °C) for 12 h, the VIGS plants were damaged to greater degree, along with significantly higher EL and MDA levels, relative to the control (Figure 5a–c). In addition, fluorescence imaging indicated that photosynthesis capacity and *F*_v_/*F*_m_ ratio were indistinguishable between the VIGS and the control plants before treatment. Exposure to the freezing temperature led to inhibition of photosynthesis and *F*_v_/*F*_m_ ratio, but the reduction was more profound in the VIGS line (Figure 5d–f). These results indicate that silencing of *PtrERF109* rendered the trifoliate orange plants more susceptible to cold. Taken together, the abovementioned data indicate that PtrERF109 is an important positive regulator of cold tolerance.

### *PtrERF109* overexpression leads to extensive transcriptional reprogramming of stress‐responsive genes

**Figure 6:**
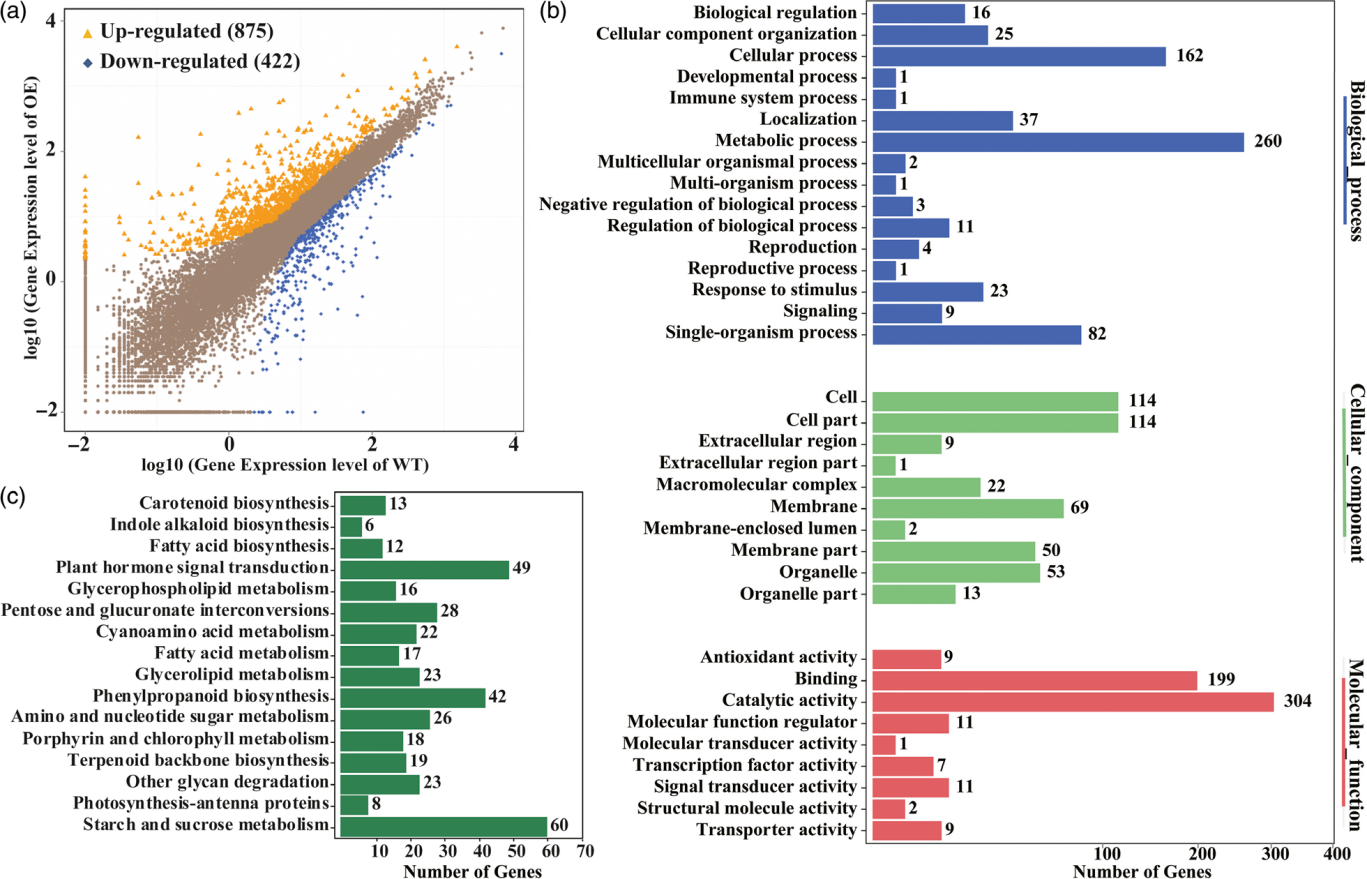
Overexpression of *PtrERF109* causes global transcriptional reprogramming in transgenic lemon. (a) Scatterplots of gene expression patterns in the transgenic line (OE) compared with the wild type (WT). Yellow triangles represent up‐regulated genes, while blue rhombuses represent down‐regulated ones. (b) GO analysis of the differentially expressed genes (DEGs). The DEGs were annotated based on biological process, molecular function and cellular component. (c) KEGG pathways enriched among the DEGs.

To gain deeper insight into the molecular mechanism underlying the enhanced cold tolerance and to identify potential target genes that may be regulated by PtrERF109, we performed RNA‐Seq using a transgenic lemon line and WT. After filtering, about 24 million clean reads were scored in each genotype (Table S1). A total of 1297 genes showed altered transcript levels (fold change ≥ 2, FDR < 0.05), 875 up‐regulated and 422 down‐regulated, in the transgenic line compared with the WT (Figure 6a; Table S2). To confirm RNA‐Seq results, qPCR analysis was performed to analyse the expression of 14 differentially expressed genes (DEGs), including nine up‐regulated and five down‐regulated genes. As can be seen in Figure S6, qPCR analyses on expression patterns for all of the tested genes were highly consistent with the fold changes revealed by RNA‐Seq, with a good correlation, suggesting that the DEG screening based on RNA‐seq is reliable.

Gene ontology (GO) analysis showed that GO terms of the DEGs at the molecular function level were primarily related to ‘catalytic activity’, ‘binding’, ‘transporter activity’, ‘signal transducer activity’ and ‘antioxidant activity’ (Figure 6b). The principal enriched biological processes of the DEGs are metabolic process, cellular process, single‐organism process, localization, cellular component biogenesis and response to stimulus. We noticed that a group of genes that have been shown to play direct roles in stress tolerance were up‐regulated in the transgenic line, including proline‐rich proteins, low‐temperature induced protein, glycine‐rich cell wall structural protein, auxin‐responsive proteins and fatty‐acid desaturase gene. In addition, we also found that an array of transcription factors that are known as positive regulators of stress response were induced in the transgenic line, including various members of AP2/ERFs (SHN1, ERF003, RAV2), MYBs (MYB15), WRKYs (WRKY46), bZIP (bZIP61) and Zinc finger proteins (ZAT10; Table S3). At cellular component level, the DEGs were mainly involved in ‘cell’, ‘cell part’, ‘membrane’, ‘membrane part’ and ‘organelle’.

The most highly enriched KEGG pathways are starch and sucrose metabolism, followed by phenylpropanoid biosynthesis, plant hormone signal transduction and lipid metabolism (Figure 6c). It is worth mentioning that several genes involved in sugar metabolism were up‐regulated in the transgenic line, including *GDSL esterase/lipase*,* Pectin methyl‐esterase inhibitor*,* UDP‐glucuronate 4‐Epimerase*, β*‐Glucosidase*,* Glycosyl transferase*,* Pectin methylesterase*,* Sucrose synthase*,* Fructokinase* and β*‐Fructofuranosidase*.

### PtrERF109 directly binds to and activates the promoter of *PtrPrx1*

**Figure 7:**
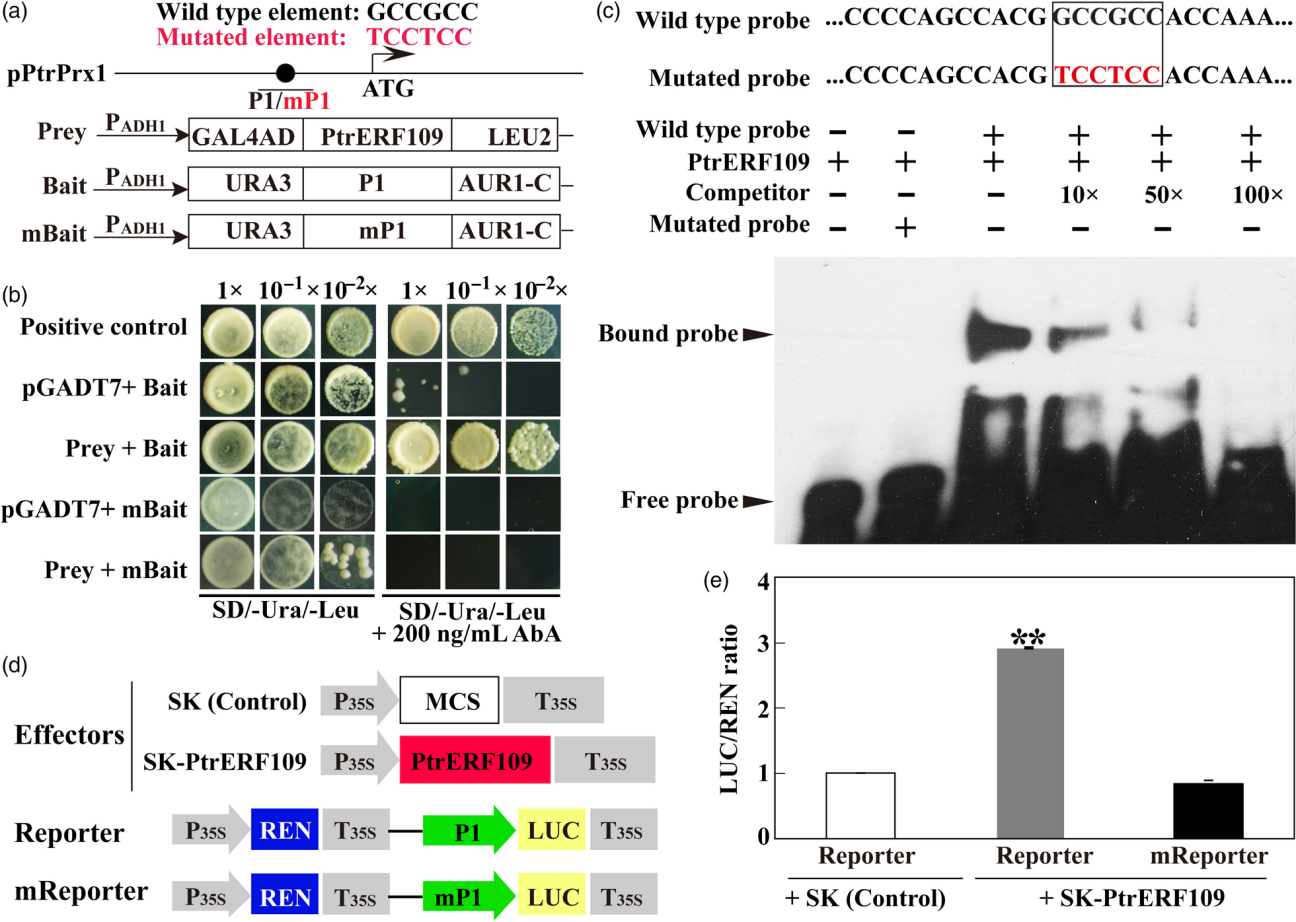
PtrERF109 binds to and activates the promoter of *PtrPrx1*. (a) Schematic diagrams of *PtrPrx1* promoter (pPtrPrx1) and constructs used for yeast one‐hybrid assay. P1 indicates the promoter fragment containing a GCC‐box sequence used for bait construction, while mP1 is a mutated form of P1 by changing ‘GCCGCC’ into ‘TCCTCC’. (b) Growth of yeast cells co‐transformed with the prey and bait, the negative control (pGADT7+bait), or the positive control (pGADT7‐Rec‐p53+p53‐AbAi) on selective medium added with or without 200 ng/mL Aureobasidin A (AbA). (c) EMSA assay of specific binding of PtrERF109 to the GCC‐box sequence in pPtrPrx1. Purified His‐PtrERF109 fusion protein was incubated with a biotin‐labelled wild type or mutated probe, along with or without the unlabelled competitor DNA. −, absence; +, presence. (d) Schematic diagrams of the effector and reporter constructs used for transient luciferase (LUC) assays. Full‐length CDS of *PtrERF109* was inserted into pGreen II 62‐SK to get an effector, while P1 or mP1 was inserted into pGreen II 0800‐LUC to generate reporters. MCS, multiple cloning site. P35S and T35S, the promoter and terminator of CaMV *35S*, respectively. REN (*Renilla* luciferase) was used as an internal control for activity normalization. (e) Transient expression assay of the promoter activity, shown as LUC/REN ratio, using tobacco protoplasts co‐transformed with the effector and the reporters. LUC/REN ratio of the Control co‐transformed with the reporters and the empty effector vector (pGreen II 62‐SK) was set as 1. Error bars represent ± SE (*n* = 3). Asterisks indicate that significant difference (**P < 0.01).

We found that five genes coding for peroxidase (orange1.1t02045, Cs7g13530, Cs6g20170, Cs2g21820, orange1.1t02036) were enriched in the GO term ‘antioxidant activity’, among which *Prx1* (orange1.1t02045) is one of the most significantly up‐regulated DEGs in the transgenic line. We are thus curious to know whether trifoliate orange *Prx1*, named as *PtrPrx1* hereafter, is a direct target gene of PtrERF109. To address this issue, we isolated the promoter sequence spanning 2000 bp upstream of the first ATG of *PtrPrx1*. A number of canonical *cis*‐acting elements associated with abiotic stress response are predicted in the promoter region, including a GCC‐box element (GCCGCC, −673 to −678 bp) that has been shown to be recognized by ERFs (Fujimoto *et al*., 2000). To demonstrate the binding of PtrERF109 to *PtrPrx1* promoter (pPtrPrx1), yeast one‐hybrid (Y1H) was performed using PtrERF109 as a prey, and a 359‐bp promoter fragments containing either original or mutated (TCCTCC) GCC‐box was used to generate baits (Figure 7a). The results demonstrate that yeast cells, regardless of the combination of prey and bait, grew well on SD/‐Ura/‐Leu medium. Nevertheless, in the presence of 200 ng/mL AbA (Aureobasidin A) only the yeast cells co‐transformed with the prey and bait containing non‐mutated GCC‐box grew normally, suggesting that PtrERF109 could bind to the GCC‐box element in the *PtrPrx1* promoter (Figure 7b).

To confirm specific binding of PtrERF109 to the GCC‐box core sequence, an electrophoretic mobility shift assay (EMSA) was carried out. When purified His‐PtrERF109 fusion protein was incubated with the wild‐type probe, a DNA‐protein complex with reduced migration was detected, whereas this complex was reduced when the unlabelled competitor was added, in a dose‐dependent manner. In addition, formation of the DNA‐protein complex was completely abolished when the GCC‐box was mutated (Figure 7c), indicating that PtrERF109 binds directly and specifically to the GCC‐box element.

We subsequently want to determine whether PtrERF109 activated *PtrPrx1* promoter *in vivo* through a dual luciferase (LUC) assay in tobacco protoplasts by using PtrERF109 as an effector and two reporters constructed with P1 containing the wild‐type GCC‐box and mP1 containing the mutated one (Figure 7d). We found that the co‐transformation of the effector and the wild‐type reporter significantly elevated the promoter activity, as revealed by the LUC/REN ratio, whereas the activity was resumed to the control level if the GCC‐box core sequence was mutated (Figure 7e), indicating that PtrERF109 activated the PtrPrx1 promoter through interacting with the GCC‐box element.

### *Prx1* expression, POD activity and ROS accumulation in transgenic and VIGS lines

**Figure 8:**
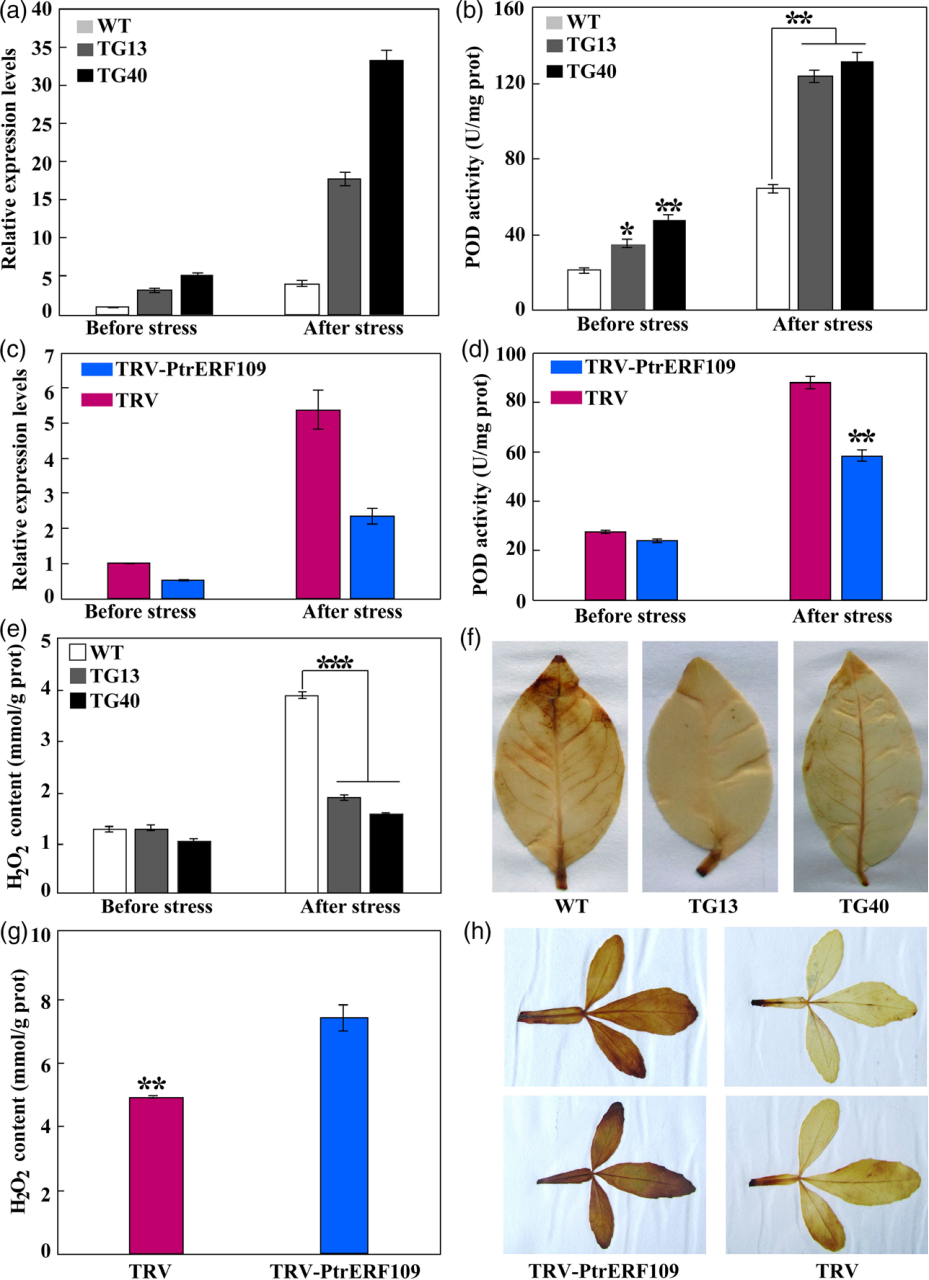
Analysis of *Prx1* gene expression, POD activity and H_2_O_2_ levels in transgenic and VIGS plants. (a–d) Relative expression level of *Prx1* (a, c) and POD activity (b, d) in lemon wild type (WT) and transgenic plants (a, b) and trifoliate orange VIGS (TRV‐PtrERF109) and TRV control plants (c, d) before and after cold treatment. (e) Levels of H_2_O_2_ in lemon transgenic and WT before and after cold treatment. (f) Levels of H_2_O_2_ in trifoliate orange VIGS and control plants after the cold treatment. (g, h) Histochemical staining with DAB for *in situ* accumulation of H_2_O_2_ in lemon transgenic and WT plants (g) and trifoliate orange VIGS and control plants (h) after the cold treatment. Error bars represent ± SE (*n* = 3). Asterisks indicate significant differences between transgenic lemon and WT or between the VIGS and control plants under the same growth conditions (*P < 0.05; **P < 0.01; ***P < 0.001).

Since *PtrPrx1* is a direct target gene of PtrERF109, it is thus assumed that ROS homoeostasis might be altered when *PtrERF109* was overexpressed or knocked down. To verify this hypothesis, we detected *Prx1* expression levels and POD activity in the transgenic or VIGS plants. As expected, the two transgenic lemon lines exhibited prominently higher expressional levels of *Prx1* and greater POD activities when compared with the WT irrespective of cold treatment (Figure 8a, b). On the contrary, when *PtrERF109* was knocked down, transcript levels of *Prx1* and activities of POD were drastically decreased in the VIGS plants (Figure 8c, d). These results indicate that overexpression of *PtrERF109* elevated, while silencing of *PtrERF109* decreased*,* the *Prx1* expression and POD activity.

It is well known that peroxidase plays a key role in scavenging H_2_O_2_, one of the major ROS, which prompts us to examine H_2_O_2_ accumulation in the tested genotypes. Under normal growth conditions, no marked difference in H_2_O_2_ levels was detected between transgenic lemon lines and the WT. However, upon exposure to the cold stress the ROS levels in the lemon transgenic lines were significantly lower than in the WT (Figure 8e). In contrast, the VIGS line accumulated more H_2_O_2_ relative to the control in the presence of cold (Figure 8f). Histochemical staining with DAB (diaminobenzidine) confirmed the difference in H_2_O_2_ levels between the tested lines after cold treatment (Figure 8g, h). Taken together, these results demonstrated that ROS accumulation was alleviated in the overexpressing transgenic lines, but enhanced in the VIGS plants following cold treatment.

### Inhibition of POD compromises cold tolerance of transgenic lemon

**Figure 9:**
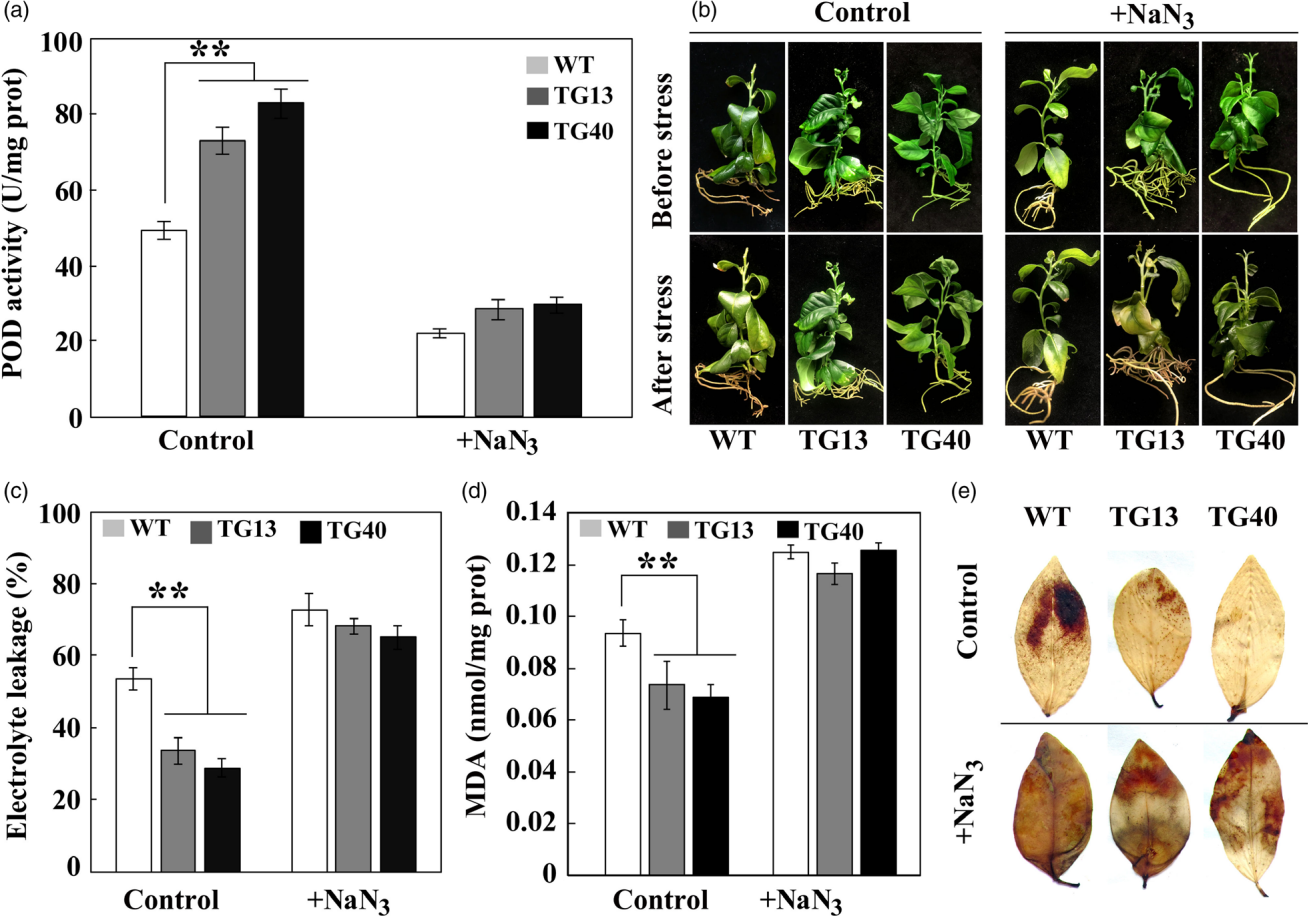
Treatment with NaN_3_ compromised cold tolerance of transgenic lemon. (a) POD activities in lemon transgenic and wild type (WT) plants treated without (Control) or with NaN_3_. (b–e) Phenotypes (b), electrolytic leakage (c), MDA content (d) and ROS staining with DAB (e) of Control or NaN_3_‐treated transgenic and WT plants before and after exposure to freezing treatment (−2 °C for 24 h). Error bars represent ± SE (*n* = 3). Asterisks indicate significant differences between the transgenic lines and the WT (**P < 0.01).

Since it takes an extremely long time to testify the role of *Prx1* in cold tolerance, we employed another approach to understand the implication of POD in cold tolerance by treating the transgenic lemon lines with NaN_3_, a potential inhibitor that has been reported to suppress POD (Zhan *et al*., 2003). NaN_3_ treatment substantially decreased endogenous POD activity in both transgenic and WT lemon in comparison with the control (without NaN_3_ treatment), and negligible difference was observed between NaN_3_‐treated transgenic lines and WT (Figure 9a). As expected, in the absence of NaN_3_ treatment the transgenic lines showed enhanced freezing tolerance relative to WT when they were exposed to −2 °C for 1 day, as revealed by plant phenotype observation, measurement of EL and MDA. However, when NaN_3_‐treated plants were subjected to the freezing conditions, the transgenic lines were damaged to the similar degree as the WT did, concurrent with nearly equivalent levels of EL and MDA in these lines (Figure 9b‐d). DAB staining showed that transgenic leaves were stained to a stronger degree compared with those without NaN_3_ treatment, a pattern close to that of the WT, implying that ROS accumulation of NaN_3_‐treated transgenic and WT lines was comparable between each other (Figure 9e). These data indicate that reduction of POD activity by NaN_3_ treatment greatly impaired cold tolerance of the transgenic plants, implying that POD might play a pivotal role in cold tolerance.

### Altered oxidative stress tolerance in transgenic and VIGS lines

**Figure 10:**
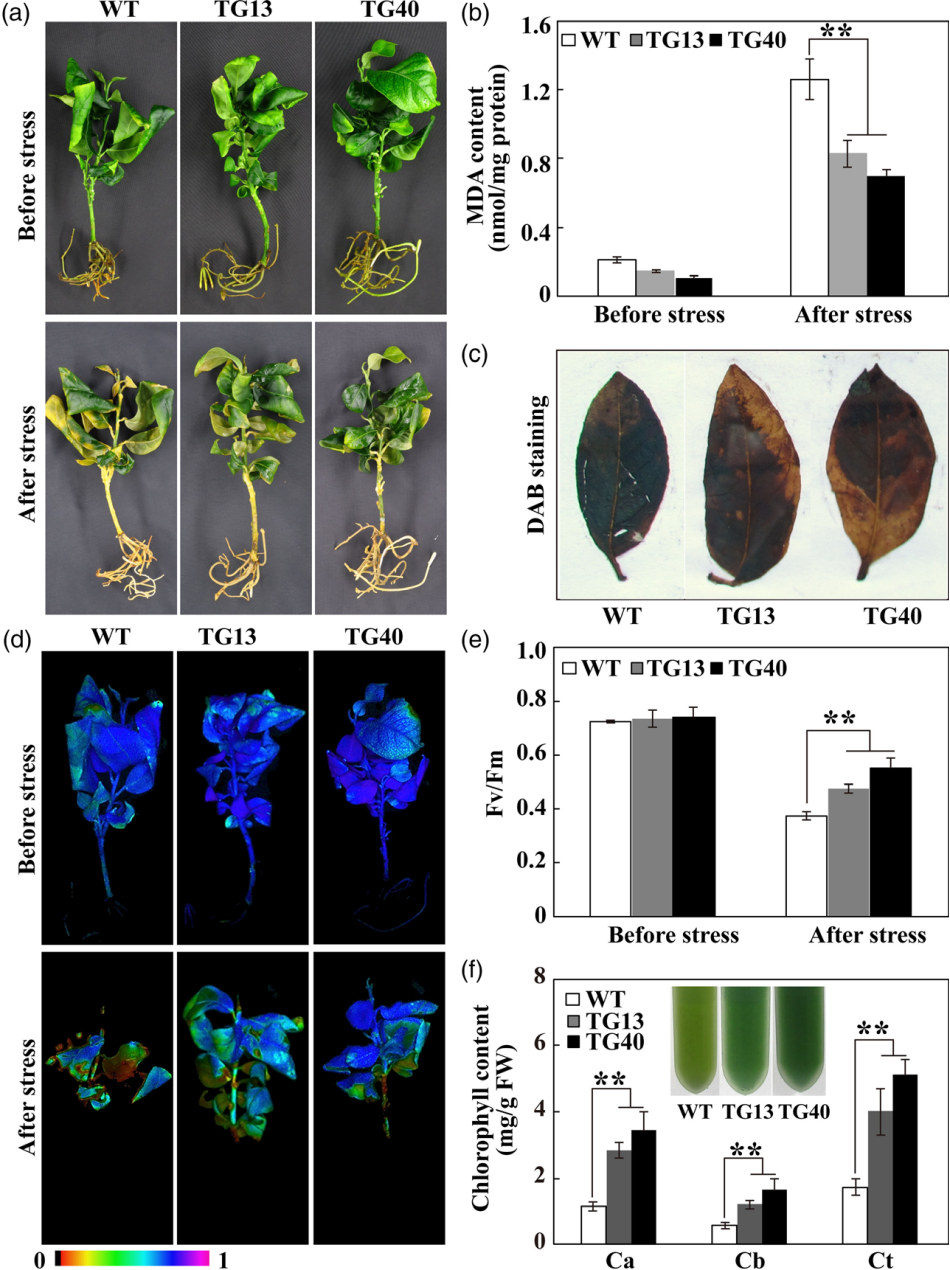
Enhanced oxidative stress tolerance in transgenic lemon plants overexpressing *PtrERF109*. (a, b) Phenotype (a) and MDA contents (b) of lemon wild type (WT) and transgenic plants before and after treatment with 100 mm H_2_O_2_ for 2 day. (c) *In situ* accumulation of H_2_O_2_ in WT and transgenic leaves treated with H_2_O_2_, as detected by DAB staining. (d, e) Chlorophyll fluorescence imaging (d) and *F*_v_/*F*_m_ ratios (e) in WT and transgenic plants before and after H_2_O_2_ treatment. (f) Chlorophyll content in H_2_O_2_‐treated WT and transgenic plants. Error bars represent ± SE (*n* = 3). Asterisks indicate significant differences between transgenic lemon and WT under the same growth conditions (**P < 0.01).

Since POD activity was altered in the overexpressing and VIGS plants, the raised question is whether oxidative stress tolerance is correspondingly modulated in these genotypes. To answer this question, transgenic lemon and WT plants were treated with exogenous H_2_O_2_. After H_2_O_2_ treatment for 2 day, leaf bleaching and chlorosis was observed, but the WT was more damaged to greater extent (Figure 10a). Although H_2_O_2_ treatment led to drastic increase of MDA contents in both genotypes, MDA levels in the *PtrERF109‐*overexpressing plants were significantly lower relative to the WT (Figure 10b). DAB staining revealed that the WT accumulated more H_2_O_2_ in comparison with the transgenic lines (Figure 10c). Chlorophyll fluorescence was prominently impaired by H_2_O_2_ treatment; however, the transgenic lines displayed better imaging, significantly higher *F*_v_/*F*_m_ ratios and chlorophyll contents than WT (Figure 10d–f).

**Figure 11:**
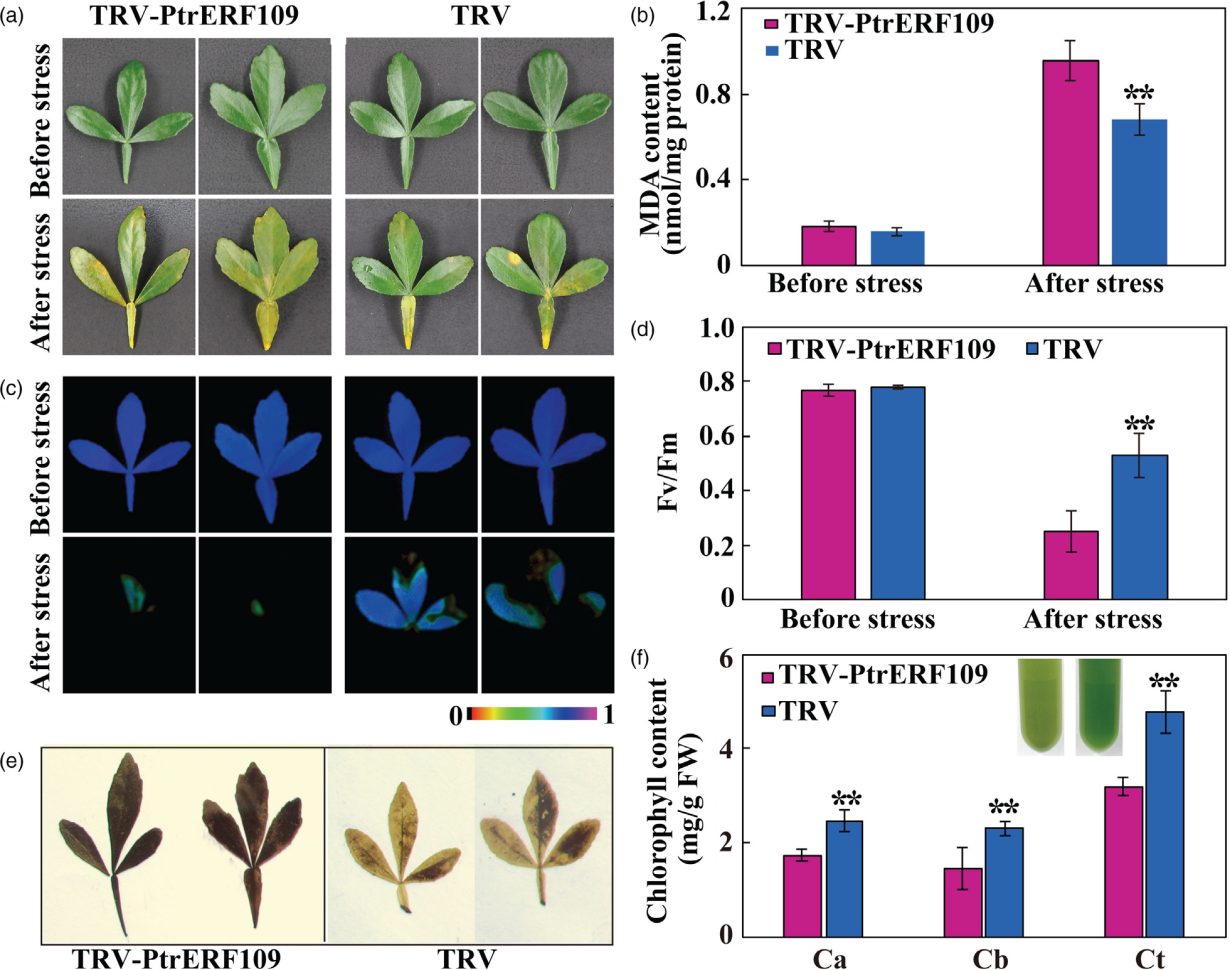
Silencing of *PtrERF109* leads to compromised oxidative stress tolerance of the trifoliate orange VIGS plants. (a‐d) Leaf morphology (a), MDA contents (b), chlorophyll fluorescence imaging (c) and *F*_v_/*F*_m_ ratios (d) of the VIGS (TRV‐PtrERF109) and control (TRV) plants before and after treatment with 200 mm H_2_O_2_ for 4 day. (e, f) *In situ* accumulation of H_2_O_2_ (e) and chlorophyll content (f) in H_2_O_2_‐treated VIGS and control leaves. Error bars represent ± SE (*n* = 3). Asterisks indicate significant differences between the VIGS and control leaves under the same growth conditions (**P < 0.01).

Upon exposure to the H_2_O_2_ treatment for 4 day, the VIGS plants exhibited more serious leaf chlorosis, significantly higher MDA level, worse chlorophyll fluorescence and lower *F*_v_/*F*_m_ ratio in comparison with the TRV control (Figure 11a–d). Meanwhile, the leaves of VIGS plants accumulated more H_2_O_2_, as shown by DAB staining, but lower chlorophyll relative to the TRV control following H_2_O_2_ treatment (Figure 11e, f). These results indicate that oxidative stress tolerance was enhanced in the *PtrERF109‐*overexpressing lines, but decreased when *PtrERF109* was silenced.

## Discussion

In this study we found that *PtrERF109* was prominently up‐regulated by cold, and that expression levels of *PtrERF109* were substantially higher relative to its counterpart in lemon under cold stress. These findings suggest that *PtrERF109* may play important roles in the cold signal transduction. Consistent with this observation, overexpression of *PtrERF109* resulted in noticeable improvement of cold tolerance, whereas silencing of *PtrERF109* led to cold susceptibility, indicating that PtrERF109 is a positive regulator of cold tolerance. In addition, we found that dehydration and salt caused either up‐regulation or down‐regulation of *PtrERF109*; whether *PtrERF109* imparts tolerance to these abiotic stresses remains to be investigated in the future. It has to be pointed out that different from the cold treatment, the salt and dehydration treatments are rather dramatic. Therefore, more moderate stress conditions are required in the future to analyse the gene expression so as to extend results and conclusions to the actual situations in the field.

Although phylogenic analysis based on the DNA binding domains indicated that PtrERF109 was most closely related to AtERF109 of Arabidopsis, PtrERF109 shared only 36.1% sequence similarity with AtERF109 at the full‐length amino acid level. Furthermore, *AtERF109* was not induced by cold according to previous report (Lee *et al*., 2005). Based on these differences, we conclude that ERF109 of different plant species may exhibit diverse functions in cold stress response. Furthermore, *PtrERF109* mRNA abundance was shown to be quickly up‐regulated by ethylene. Ethylene plays important roles in regulating responses to a wide variety of external environmental stimuli, and ERFs are crucial components for relaying the ethylene signalling (Yang *et al*., 2015). Several ERFs have been previously reported to be induced by ethylene, such as *JERF1* (Zhang *et al*., 2004), *OsERF1* (Hu *et al*., 2008b), and *VaERF057* (Sun *et al*., 2016), suggesting that these genes may be implicated in the ethylene signalling pathway to orchestrate stress tolerance.

TFs are known to function in plant stress responses via regulating downstream target genes (Nakashima *et al*., 2009). In this study, we undertook a global transcriptome analysis by RNA‐Seq and found that overexpression of *PtrERF109* led to a comprehensive transcriptional reprogramming. We noticed that transcript levels of 44 TFs were altered in the overexpressing line, suggesting that the regulatory cascade of these TFs is possibly modulated by PtrERF109. In addition, several crucial metabolic pathways were prominently influenced by overexpression of *PtrERF109*. One of them is related to carbohydrate metabolism, as 113 DEGs related to sugar biosynthesis, transportation and degradation were enriched in the transcriptome. Some of them, including β*‐D‐xylosidase*,* UDP‐glucose epimerase*,* Glycosyl transferase* and *Sucrose synthase*, were up‐regulated in the transgenic line. It has been documented that soluble sugars represent not just an energy source but also serve as cryoprotectants, metabolites exhibiting antioxidative activity and signalling molecules (Dahro *et al*., 2016; Nakabayashi and Saito, 2015; Peng *et al*., 2014). Therefore, *PtrERF109* overexpression may lead to a substantial change in gene expression profiles involved in the sugar metabolism to prepare enough carbohydrate for combating with the environmental cues. Another pathway that draws our attention is the lipid metabolism. It is well known that remodeling of membrane fluidity is required for plants to adapt to cold stress and that fatty acid desaturases (FAD) plays a crucial role in modification of membrane fluidity by modulation of unsaturated fatty acid levels (Upchurch, 2008). Increasing studies showed that there is a correlation between expression levels of *FAD* gene and cold tolerance (Dominguez *et al*., 2010; Shi *et al*., 2012). Herein, we found several *FAD* genes were substantially up‐regulated in the transgenic line, implying that overexpression of *PtrERF109* might lead to an increase in the unsaturated fatty acid levels, thus promoting remodelling of the membrane fluidity. Lastly, but not the least, the phenylpropanoid biosynthesis is noticeably enriched in the transgenic line. Abiotic stresses have been reported to activate multiple branches of the phenylpropanoid pathway, which is tightly responsible for production of a variety of specialized secondary metabolites, such as lignins, isoflavonoid phytoalexins, phenolic compounds and flavonoids, which have been previously shown to act as an adaptation mechanism for plants to cope with abiotic cues (Nakabayashi and Saito, 2015; Savoi *et al*., 2016). Taken together, fine‐tuning of these metabolic pathways, along with others not discussed here, may be attributed to the enhanced cold tolerance in the transgenic lines overexpressing PtrERF109.

It is known that ROS, at high levels, are toxic to plant cells by damaging nucleic acids, oxidizing proteins and causing lipid peroxidation, representing a major factor responsible for cell viability under abiotic stresses (Gill and Tuteja, 2010). Under steady state conditions there is a delicate equilibrium between ROS production and scavenging. However, the equilibrium is perturbed by abiotic stresses, leading to increase of intracellular ROS levels (Gill and Tuteja, 2010). It is well established that antioxidant enzymes, such as superoxide dismutase (SOD), POD and catalase (CAT), play a crucial and predominant role in eliminating the ROS produced under abiotic stresses (Gill and Tuteja, 2010). Increasing evidence demonstrated that a high antioxidant capacity to scavenge the ROS is linked to increased tolerance to environmental stresses (Geng and Liu, 2018; Huang *et al*., 2013). Interestingly, we found that several POD‐encoding genes were considerably up‐regulated in the *PtrERF109*‐overexpressing line. Of note, *Prx1* was the most highly up‐regulated one. Interestingly, transcript levels of *Prx1* and POD activities of the transgenic lines were significantly higher than those of the WT. In contrast, *Prx1* levels and POD activities were decreased in the VIGS line with silenced *PtrERF109*, particularly under cold stress. These results demonstrate that the transgenic plants display more robust antioxidant capacity relative to the WT, which was supported by remarkable reduction of ROS levels and greater tolerance to oxidative stress in the transgenic overexpressing lines, whereas the VIGS line exhibited opposite trend. Therefore, more efficient mobilization of the antioxidant system, leading to enhanced antioxidation capacity, is another mechanism underlying the positive role of *PtrERF109* in cold tolerance. The greater antioxidant capacity in the overexpressing lines was supported by oxidative stress tolerance assay using H_2_O_2_ treatment. However, it is worth mentioning that the effect of H_2_O_2_ treatment was drastic and the conditions were rare in nature, which is not a physiological condition that plants face in the field.

It is known that the ERFs participate in transcriptional regulation through binding to the GCC‐box element within the promoters of target genes (Licausi *et al*., 2013). To our surprise, although transcript levels of the antioxidant genes were higher in lemon transgenic lines, GCC‐box core sequence was only observed in the promoter of *PtrPrx1*. This raises the question of why and how the other antioxidant genes were up‐regulated by overexpression of *PtrERF109*. One possibility is that PtrERF109 may indirectly regulate these genes via some unidentified intermediate transcription factors. Such assumption is not impossible as a number of TFs were shown to be up‐regulated in the global transcriptome profiling. In contrast, *PtrPrx1* might be under the direct control of PtrERF09 since PtrERF109 can bind to and activate the promoter of *PtrPrx1*, indicating that *PtrPrx1* is a direct target of PtrERF09. This result agrees well with the elevation or reduction of *Prx1* mRNA abundance in the overexpressing and VIGS line, respectively. So far, a plethora of target genes have been characterized for various ERF members. For instance, *CaPF1* was proposed to regulate stress‐related genes, such as *PR* and *COR* (Yi *et al*., 2004). In another study, TaPIE1 was reported to mediate responses to pathogen attack and freezing stresses by regulating the stress‐related genes, including *PR10*,* P5CR*,* ICE1* and *POX2* (Zhu *et al*., 2014). Interestingly, AtERF1 was shown to regulate specific suites of genes, including *b‐CHI*,* PDF1.2*,* ELI3‐2*,* GEA6*,* LEA4‐5* and *HSP70*, in a stress type‐dependent manner (Cheng *et al*., 2013). However, direct regulation of a *POD* gene by ERFs has never been reported. Herein, characterization of *PtrPrx1* as a direct target of PtrERF109 provides valuable clues to better understanding of the ERF regulon associated with abiotic stress response. It may gain new insight into the regulatory cascade with respect to activation of the antioxidant genes, which has been widely observed in diverse plants exposed to cold or other stresses (Kim *et al*., 2012; Shigeto and Tsutsumi, 2016). In addition, we found that some known ERF target genes were also found in our RNA‐Seq data. These include a sugar transporter gene *SWEET* (Cs3g20720), which was reported to be directly regulated by a waterlogging‐responsive ERF from *Menthain* (Phukan *et al*., 2018), and a CaM‐like protein (CML) gene (Cs5g22810), a direct target of OsERF48 involved in drought tolerance (Jung *et al*., 2017). These findings suggest that ERFs from various plant species may regulate a set of common target genes for coping with the environmental stresses.

## Conclusion

In summary, a cold‐responsive ERF member PtrERF109 from *Poncirus trifoliata* function as a positive regulator of cold tolerance. Overexpression of *PtrERF109* led to extensive alteration of the global transcriptome, leading to reprogramming of an array of genes involved in various metabolic pathways and antioxidant machinery. In particular, the POD‐encoding gene *PtrPrx1* was shown to be directly targeted by PtrERF109 through interacting with the GCC‐box core sequence. Taken together, modulation of ROS homoeostasis via directly regulating the ROS‐scavenging gene is responsible, at least in part, for the role that PtrERF109 plays in cold tolerance.

## Experimental procedures

### Plant materials and stress treatments

Seeds of trifoliate orange (*Poncirus trifoliata*) and lemon collected from a nursery at Huazhong Agricultural University were sown in soil pots and germinated under 16 h light/8 h dark photoperiod at 25 °C. Three‐month‐old seedlings were used to examine expression patterns of *PtrERF109* under various treatments, including cold, dehydration, salt and ethylene treatments. For cold treatment, the seedlings were kept in a plant growth chamber set at 4 °C for 0, 1, 3, 6, 12, 24 and 48 h. For dehydration treatment, the trifoliate orange seedlings were air‐dried on filter papers at ambient temperature for 0, 0.5, 1, 3, 5 and 7 h. For salt treatment, the seedlings were placed in conical flasks containing 250 mm NaCl for 0, 3, 6, 12, 24 and 48 h. For ethylene treatment, the seedlings were placed in an airtight box added with 10 mm ethephon for 0, 1, 3, 6, 12, 24 and 48 h. In another experiment aiming to compare expression patterns of *PtrERF109* and *ClERF109* in response to cold treatment, seedlings of trifoliate orange and lemon were placed at 4 °C for 0, 6, 24 and 72 h. Leaves were sampled at the indicated time points, immediately frozen in liquid nitrogen and stored at −80 °C until further analyses.

### RNA extraction and quantitative real‐time PCR analysis

Total RNA was extracted using a Trizol reagent (Takara) and treated with DNase I (Thermo) to remove genomic DNA. Synthesis of cDNA was conducted using RevertAid First Strand cDNA synthesis Kit (Thermo). Quantitative real‐time PCR (qPCR) was performed on an Applied Biosystems 7500 Real‐Time PCR system using the SYBR Green Master Mix (Qiagen) according to the user manual. Each reaction contained 5 μL of SYBR Green Master Mix, 0.05 μL of QN ROX Reference Dye, 100 ng cDNA and 1 μm forward and 1 μm reverse primers in a final volume of 10 μL. The PCR cycling regimes were composed of a denaturation step of 95 °C for 2 min, followed by 45 cycles of 95 °C for 10 s, 60 °C for 30 s and 72 °C for 25 s. Relative gene expression levels were detected using the 2^−ΔΔCT^ algorithm (Livak and Schmittgen, 2001) by normalizing to expression of the *Actin* gene, which was used as an internal reference control and analysed in parallel. Primer sequences are listed in Table S4 (unless otherwise stated, all primers are shown in this table). Three technical replicates were used for each sample and the data are shown as means ± SE (standard errors).

### Histochemical assay of GUS activity

Histochemical assay of GUS (β‐glucuronidase) activity was carried out using transient expression in sweet orange (*Citrus sinensis*) embryogenic callus. For this purpose, the *PtrERF109* promoter (pPtrERF109, 1973 bp DNA fragment upstream of the translation start site) was amplified from trifoliate orange genomic DNA based on the genome sequence of its homologous gene in sweet orange (Cs8g05910) and inserted into DX2181 vector containing a *GUS* gene to generate pPtrERF109:GUS construct. The resulting construct was transformed into the callus by *Agrobacterium*‐mediated transformation as described previously (Dai *et al*., 2018). After culture at ambient temperature for 3 day in the dark, the callus was treated for 24 h at 4 °C. For histochemical staining the callus was vacuum infiltrated for 30 min in a GUS reaction buffer containing 1 mm X‐Gluc (5‐bromo‐4‐chloro‐3‐indolyl‐β‐D‐glucuronide), 0.1 mm sodium phosphate buffer (pH 7.0), 10 mm EDTA, 1 mm potassium ferricyanide, 1 mm potassium ferrocyanide and 0.5% Triton X‐100.

### Cloning and sequence analysis of *PtrERF109*

Unigene20109 was used to Blastn search against the genome of sweet orange (*Citrus sinensis*), which is closely related to trifoliate orange, to get a homologous gene, Cs8g05910. A pair of primers (Forward: 5′‐ATTCCAGAGCCAACACGAAC‐3′, Reverse: 5′‐GAACGTGGGATTTCGCCAGC‐3′) was then designed according to the sequence of Cs8g05910 and used to amplify the full‐length coding sequence (CDS) of *PtrERF109* from trifoliate orange. Multiple sequence alignments were performed using ClustalX software based on the AP2 domains of PtrERF109 and other 21 ERF subfamily genes (accession numbers are shown in Table S5) or between PtrERF109 and its homologues from citrus species and its relatives, including *C. sinensis*,* C. clementina*,* C. limon*,* C. medica*,* C. grandis*,* C. ichangensis*,* C. reticulata*,* Fortunella crassifolia* and *Atlantia buxifolia*, which were obtained from http://citrus.hzau.edu.cn/orange/index.php and https://phytozome.jgi.doe.gov. The alignment results were displayed with GENEDOC software. MEGA 7.0 was used to construct a phylogenetic tree based on the neighbour‐joining method and bootstrap analysis with 1000 replications.

### Subcellular localization analysis

To determine the subcellular localization pattern of PtrERF109, the complete open reading frame (ORF) without the stop codon was amplified and cloned into *Bam*HI and *Sma*I restriction sites of an expression vector 101LYFP containing the YFP reporter gene, under the control of cauliflower mosaic virus (CaMV) *35S* promoter, to form a construct *35S: PtrERF109‐YFP*. The fusion construct (35S:PtrERF109‐YFP) and control vector (35S:YFP) were integrated into *Agrobacterium tumefaciens* GV3101. Tobacco (*N. benthamiana*) leaves were agroinfiltrated with GV3101 carrying either the fusion construct or the control, as has been described previously (Walter *et al*., 2004). The infiltrated plants were grown for an additional two days prior to fluorescence signal detection using a laser scanning confocal microscope (Leica TCS SP8). DAPI (4′‐6‐Diamidino‐2‐phenylindole) was used to stain the nuclei.

### Transcriptional activation activity assay

For the transcriptional activation assay, full‐length ORF and two truncated (ERF109ΔC, ERF109ΔN) ORF fragments of PtrERF109 were amplified by PCR with specific primers and subcloned in fusion with GAL4‐DBD (DNA binding domain) of pGBKT7 vector (Clontech). All of the fusion constructs and empty vector were separately transformed in yeast strain *AH109*. The yeast cells were plated on synthetic dropout (SD)/‐Trp or SD/‐Trp/‐Ade/‐His medium supplemented with 0 or 10 mm 3‐AT (3‐Amino‐1,2,4‐Triazole). Activity of α‐galactosidase was examined by plating the transformants on SD/‐Trp/‐Ade/‐His medium containing X‐α‐Gal.

### Vector construction and plant transformation

The full‐length coding sequence (CDS) of *PtrERF109* was amplified by PCR with primers containing *Xba*I or *Sma*I linker and cloned into the expression vector pBI121 at the same restriction sites, driven by the CaMV *35S* promoter. The resulting plasmid was mobilized into *A. tumefaciens* strain GV3101 by heat shock. *Agrobacterium*‐mediated transformation of tobacco (*Nicotiana tabacum*) and lemon (*C. limon*) were performed according to previous methods (Fu *et al*., 2011). The transformed explants were selected on MS (Murashige and Skoog, 1962; for tobacco) or MT (Murashige and Tucker, 1969; for lemon) medium containing 50 mm kanamycin. The regenerated plants after the kanamycin selection were verified by PCR using two pairs of specific primers; only those plants yielding expected PCR amplicons were regarded as positive lines. Expression levels of *PtrERF109* in transgenic tobacco lines were examined by semi‐quantitative PCR according to Gong *et al*. (2015), whereas the transgenic lemon lines were assessed using qPCR as mentioned above. *Ubiquitin* and *Actin* were used as internal reference genes for tobacco and lemon, respectively. The stable tobacco lines at T_2_ generation and vegetatively multiplied lemon lines were used for the subsequent experiments.

### Generation of VIGS plants

The tobacco rattle virus (TRV)‐based vectors (pTRV1 and pTRV2) were used for the VIGS assessment. A 358‐bp fragment of *PtrERF109* was amplified and insert into *Bam*HI and *Sma*I sites of pTRV2 vector. The pTRV1, pTRV2 (control) and fusion constructs were separately transformed into *A. tumefaciens* strain GV3101 by heat shock. The bacterial infection suspensions were prepared as previously described (Dai *et al*., 2018), and agroinfiltration was carried out by submerging the germinating seeds of trifoliate orange harbouring with shoots about 1 cm long into the bacterial suspensions in a vacuum chamber (Dai *et al*., 2018; Yan *et al*., 2012). After vacuum infiltration, the germinating seeds were dried on filter papers and cultured in dark for three days. The seeds were rinsed with water, and then sown in soil pots, which were placed in a growth chamber (25 °C, 16 h light/8 h dark). Thirty days later, DNA of each seedling was extracted, and subjected to genomic PCR analysis using two pairs of primers for detection of positive plants, while transcript levels of *PtrERF109* in each positive plant was analysed using qPCR.

### Cold tolerance assays

Seeds of wild type (WT) and transgenic tobacco lines were sown in pots filled with a 3 : 1 mixture of soil and vermiculite, and kept at 25 °C in a growth chamber with a photoperiod of 16 h light/8 h dark at 25 °C. For cold tolerance assay, 30‐day‐old tobacco plants were exposed to −2 °C for 12 h. The transgenic lemon and wild type plants were firstly transplanted into soil and grown for about 2 months, before exposure to freezing treatment for 8 h at −4 °C. As for the VIGS plants, 1‐month‐old VIGS seedlings were exposed to −2 °C for 12 h. Survival rate, EL, MDA, ROS levels and POD activity of the tested lines were examined, while photosynthesis efficiency of the treated plants was monitored based on fluorescence imaging. In addition, transgenic and wild type lemon plants were hydroponically grown at normal temperature in 100 mm NaN_3_ (a POD inhibitor, Zhan *et al*., 2003) for 10 h, using water as a control, prior to freezing treatment at −2 °C for 24 h. Leaves harvested before and after freezing treatment were used for measurements of MDA, EL and DAB staining, while those sampled after NaN_3_ treatment were used to analyse POD activity.

### Oxidative stress tolerance assays

For assessment of oxidative stress tolerance, 1‐month‐old lemon plants (wild type and transgenic) or trifoliate orange leaves (control and VIGS) were submerged in H_2_O_2_ solution (100 mm for lemon and 200 mm for trifoliate orange) for 2 and 4 days, respectively. Photographs were taken before and after the treatments. The leaves were sampled and used for physiological measurement, including MDA levels, chlorophyll content and ROS detection. Fluorescence imaging was also conducted to analyse the photosynthesis efficiency.

### Physiological measurements and histochemical staining

The MDA and H_2_O_2_ contents and POD activities were measured using specific detection kit (A003‐1 for MDA, A064 for H_2_O_2_, A084‐3 for POD, Nanjing Jiancheng Bioengineering Institute, China) following the manufacturer's instructions. Total protein levels were determined using Coomassie Brilliant Blue G‐250 staining method according to Bradford (1976). One unit of POD activity was defined as an increase of 0.01 per min in the absorbance at 470 nm. H_2_O_2_ was also examined by histochemical staining with DAB as described by Wang *et al*. (2011). Chlorophyll fluorescence imaging was performed using an IMAGING‐PAM chlorophyll fluorimeter, and *F*_v_/*F*_m_ ratios were calculated using Imaging WinGigE software (Walz, Germany). In addition, chlorophyll was extracted using acetone and quantified according to Liu *et al*. (2008). EL was analysed according to Dahro *et al*. (2016).

### RNA‐Sequencing and analysis

For RNA‐sequencing (RNA‐Seq) analysis, leaves were sampled from 60‐day‐old plants of lemon wild type and a transgenic line, generating two samples. Two biological replicates were used for each genotype sample. RNA‐Seq and bioinformatics analyses were conducted by BGI (Shenzhen, China). Total RNA was isolated using the RNeasy Mini Kit (Qiagen) and subjected to excluding genomic contamination by treatment with DNase I (Qiagen). The cDNA libraries were constructed and sequenced on an Illumina Hiseq platform (BGI, Shenzhen, China). The library constructions were carried out following the manufacturer's instruction of NEBNext Ultra RNA Library Prep Kit for Illumina (NEB, E7530) and NEBNext Multiplex Oligos for Illumina (NEB, E7500), and were sequenced with Illumina HiSeq™ 2500 sequencing platform. After filtering, the clean reads of each sample were mapped to the sweet orange database (http://citrus.hzau.edu.cn/orange/index.php) using the HISAT and Bowtie2 softwares (Kim *et al*., 2015; Langmead *et al*., 2009). Gene expression levels were quantified as FPKM (fragments per kilobase of exon per million fragments mapped) by a software package called the RSEM software (Li and Dewey, 2011). Differentially expressed genes (DEGs) were screened by NOISeq method (Tarazona *et al*., 2015) in the light of fold changes that were calculated on the basis of FPKM. The DEGs were defined according to the following thresholds: fold change ≥2, false discovery rate (FDR) <0.05. Gene ontology enrichment analysis of the DEGs was performed for the DEGs using agriGO toolkit (Tian *et al*., 2017). In addition, KEGG (Kyoto Encyclopadia of Genes and Genomes) is used to perform pathway enrichment analysis of DEGs.

### Yeast one‐hybrid assay

The original *PtrPrx1* promoter fragment (P1, 359 bp long) containing a genuine GCC‐box element (GCCGCC) was amplified by PCR, whereas mP1 is a mutated P1 by changing its GCC‐box sequence into ‘TCCTCC’. Either P1 or mP1 was cloned into pAbAi vector as the baits, and the full‐length CDS of *PtrERF109* was fused at C terminus of GAL4‐AD in pGADT7 vector to construct the prey. Yeast one‐hybrid (Y1H) assay was carried out using the Matchmaker Gold Y1H Library Screening System (Clontech, Mountain View, CA) according to the user manual. Protein‐DNA interaction was determined based on growth ability of the co‐transformed yeast cells on SD/‐Ura/‐Leu medium supplemented with 200 ng/mL AbA following the manufacturer's protocol.

### Electrophoretic mobility shift assay (EMSA)

The ORF of *PtrERF109* was cloned into the pHMGWA vector containing a His tag and expressed in the *Escherichia coli* strain Rosetta (DE3). The recombinant His‐PtrERF109 protein was induced by 0.5 mm IPTG (isopropyl β‐D‐1‐thiogalactopyranoside) at 37 °C for 6 h and purified using the Ni‐NTA Agarose (Qiagen) according to the manufacturer's instructions. A 48‐bp single‐stranded oligonucleotide was synthesized based on P1 or mP1 sequences and labelled with biotin at the 3′‐end by Shanghai Sangon Biotechnology (Shanghai, China), while the same fragment harbouring the wild type GCC‐box without biotin labelling was used as a competitor. EMSA was performed using the LightShift Chemiluminescent EMSA Kit (Pierce). The probes were incubated with the fusion protein for 30 min at room temperature in the binding reaction (2.5% glycerol, 5 mm MgCl_2_, 50 ng/μL Poly (dI•dC), 0.05% NP‐40, 50 mm KCl, 10 mm EDTA, 1× Binding Buffer), along with or without the competitor. The protein‐bound DNA was separated from the unbound ones on 6.5% nondenaturing polyacrylamide gels and electroblotted onto a nylon membrane (Biosharp), followed by chemiluminescence detection.

### Dual LUC assays

The full‐length ORF of *PtrERF109* was fused into the pGreenII 62‐SK binary vector using the CloneExpress™ II One Step Cloning Kit (Vazyme) to generate an effector construct, while wild type or mutated promoter fragment sequences were inserted into pGreenII 0800‐LUC to generate reporters. Protoplasts isolated from tobacco (*N. benthamiana*) leaves were used for transient gene expression analysis as previously described (Yoo *et al*., 2007). The transformed protoplasts were incubated at 25 °C in dark for 16 h and then subjected to LUC assays using the Dual‐Luciferase^®^ Reporter Assay System (Promega) according to the manufacturer's instructions.

### Statistical analysis

Stress treatments were repeated at least twice with three replicates for each line. Data were analysed by SAS software package (SAS Institute, Cary, NC). Analysis of variance (ANOVA) was used to compare the statistical difference based on Fisher's least significant difference test at the significance levels of *P* < 0.05 (*), *P* < 0.01 (**) and *P* < 0.001 (***).

## Conflict of interest

The authors declare no conflict of interest.

## Supporting information


**Figure S1** Sequence alignments of AP2 domains from PtrERF109 and its homologues from other citrus species and its relatives.
**Figure S2** Expression patterns of *PtrERF109* from trifoliate orange and *ClERF109* from lemon in response to cold.
**Figure S3** Generation and identification of transgenic tobacco plants overexpressing *PtrERF109*.
**Figure S4** Generation and identification of transgenic lemon plants overexpressing *PtrERF109*.
**Figure S5** Molecular characterization of the VIGS plants by genomic PCR and qPCR.
**Figure S6** Validation of differentially expressed genes by qPCR analysis.

**Table S1** Summary of RNA‐sequencing data for the two replicates of each genotype.

**Table S2** Differentially expressed genes **(**DEGs) in transgenic lemon line.

**Table S3** Differentially expressed transcription factors in transgenic lemon line.

**Table S4** List of primers used in this study.

**Table S5** Accession numbers of the ERF genes used for phylogenetic tree construction.
