# Supplementary material for: ERF109 of trifoliate orange (Poncirus trifoliata (L.) Raf.) contributes to cold tolerance by directly regulating expression of Prx1 involved in antioxidative process
Source: Plant Biotechnol J. 2019 Jan 4;17(7):1316–32. doi: 10.1111/pbi.13056 (PMC6576027; doi:10.1111/pbi.13056)
Supplement: Supplementary file 1 — Figure S1 Sequence alignments of AP2 domains from PtrERF109 and its homologues from other citrus species and its relatives. Figure S2 Expression patterns of PtrERF109 from trifoliate orange and ClERF109 from lemon in response to cold. Figure S3 Generation and identification of transgenic tobacco plants overexpressing PtrERF109. Figure S4 Generation and identification of transgenic lemon plants overexpressing PtrERF109. Figure S5 Molecular characterization of the VIGS plants by genomic PCR and qPCR. Figure S6 Validation of differentially expressed genes by qPCR analysis. [file PBI-17-1316-s005.docx]

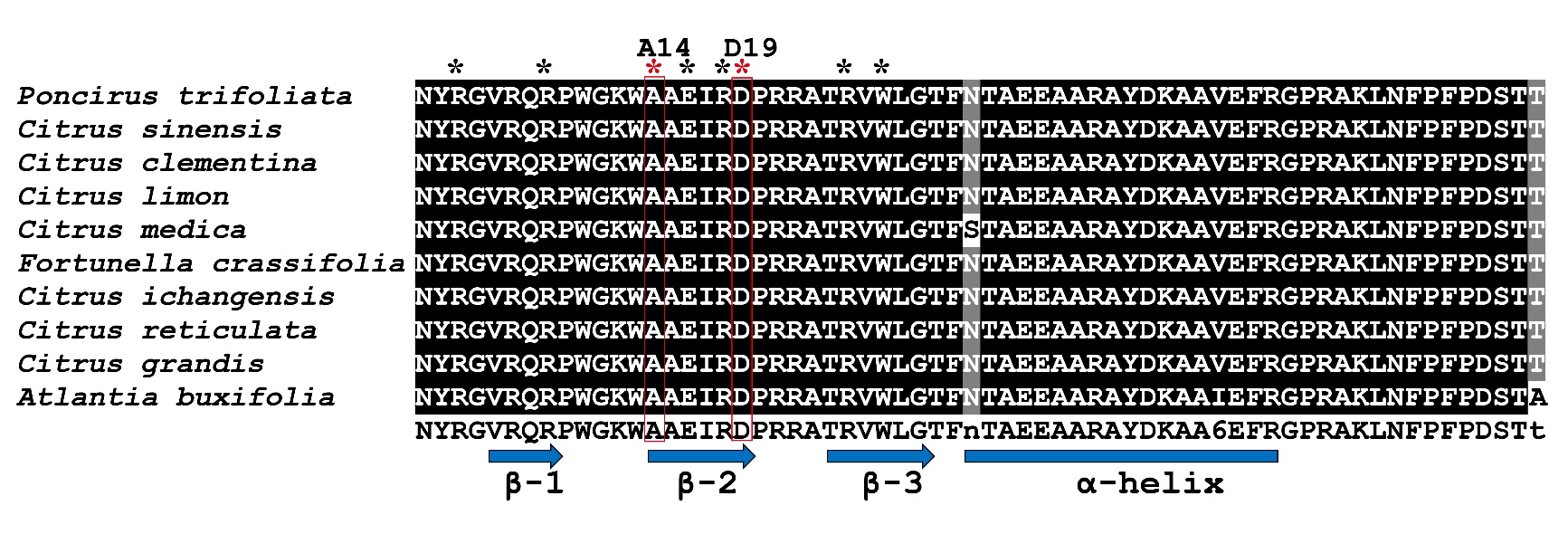


**Figure S1**. **Sequence alignments of AP2 domains from PtrERF109 and its homologues from other citrus species and its relatives**. Except PtrERF109, AP2 sequences of *Citrus sinensis*, *C. clementina*, *C. limon C. medica*, *C. grandis*, *C. ichangensis*, *C. reticulata*, *Fortunella crassifolia* and *Atlantia buxifolia* are used.


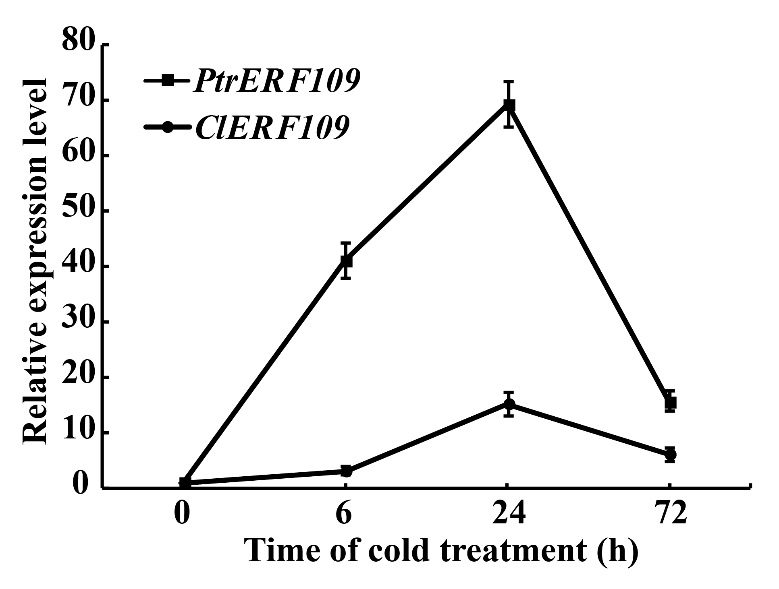


**Figure S2**. **Expression patterns of *PtrERF109* from trifoliate orange and *ClERF109* from lemon in response to cold.** *Actin* gene was used as an internal control. Error bars represent ± SE (n = 4).


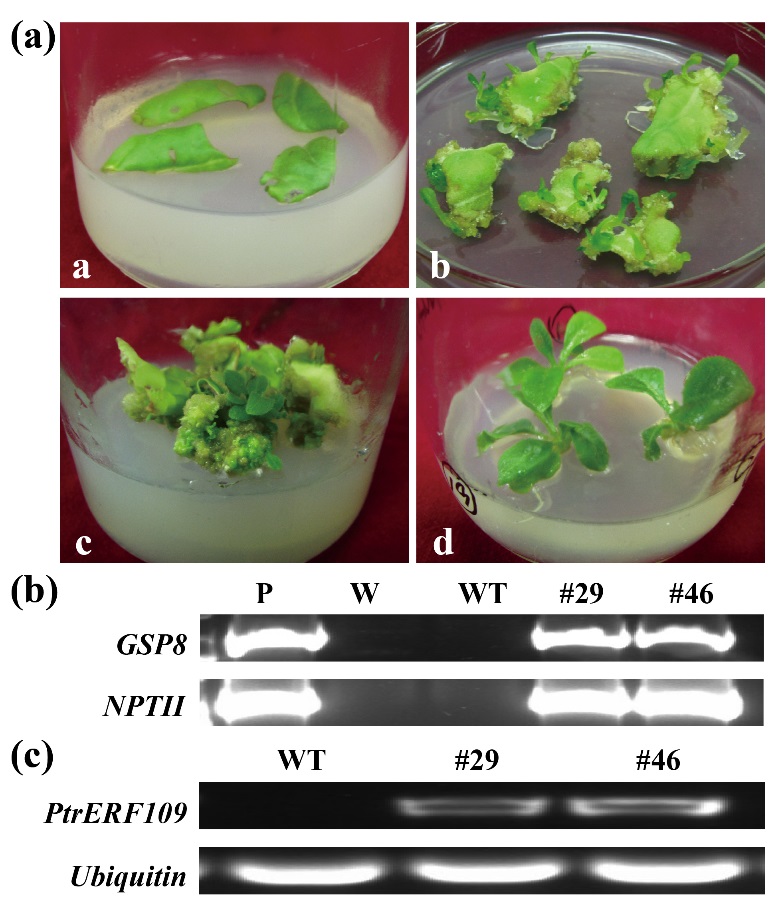


**Figure S3. Generation and Identification of transgenic tobacco plants overexpressing *PtrERF109*.** (a) Tobacco transformation and regeneration. (a)-a, Co-culture of tobacco leaf pieces; (a)-b. Growth on the selection medium for 30 d; (a)-c. Generation and multiplication of kanamycin-resistant buds; (a)-d. Rooted plants. (b) PCR identification of the regenerated buds using *GSP8* and *NPTII* (neomycin phosphotransferase II) primers on the upper panel and lower panel, respectively. P, plasmid; W, ddH_2_O; WT, wild type; #29 and #46, transgenic lines. (c) Expression analysis of *PtrERF109* in transgenic and WT tobacco by semi-qPCR. *Ubiquitin* was used as an internal control.


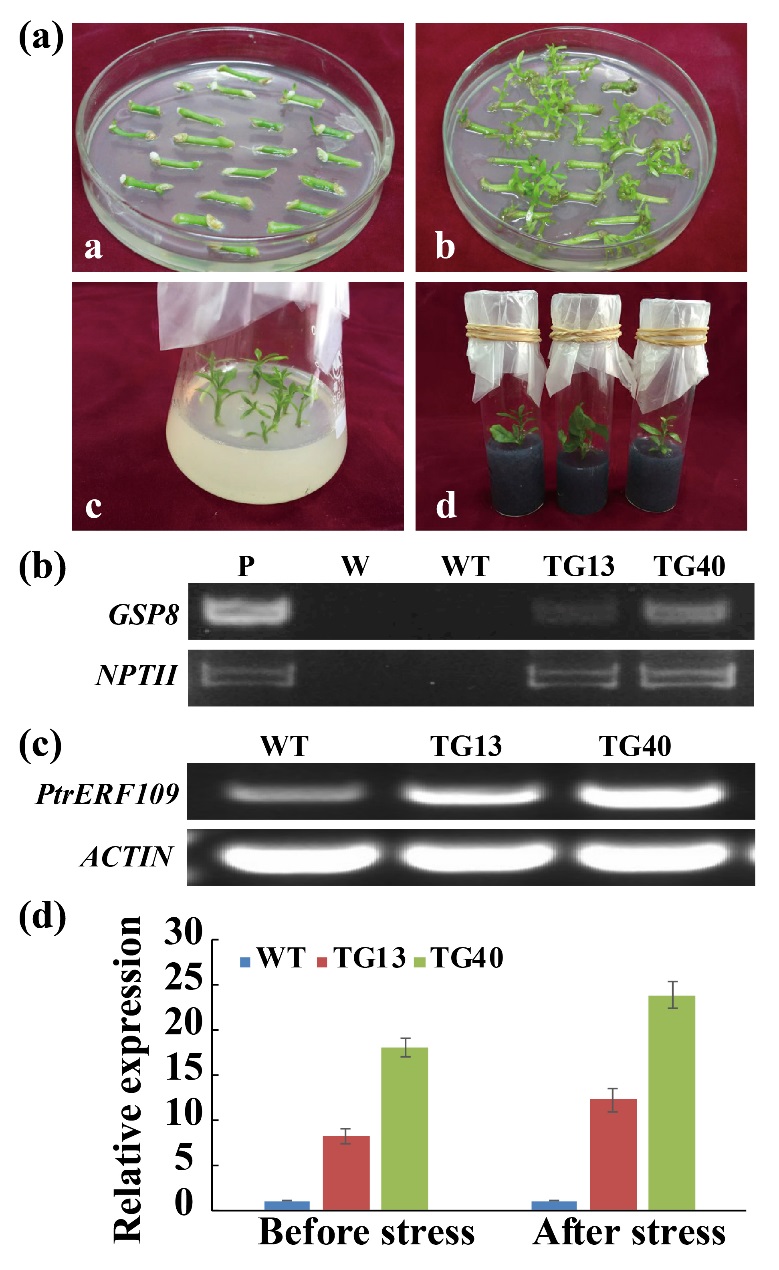


**Figure S4. Generation and Identification of transgenic lemon plants overexpressing *PtrERF109*.** (a) Lemon transformation and regeneration. (a)-a, Co-culture of lemon shoot segments; (a)-b. Growth on the selection medium for 40 d; (a)-c. Elongation and multiplication of kanamycin-resistant shoots; (a)-d. Rooted plants. B, PCR identification of the plants using specific primers of *GSP8* and *NPTII* primers. P, plasmid; W, ddH_2_O; WT, wild type; TG13 and TG40, transgenic lines. (c, d) Expression analysis of *PtrERF109* in transgenic lines by semi-qPCR (c) and qRT-PCR (d), respectively. *Actin* was used as an internal control. Error bars indicate ± SE (n = 4).


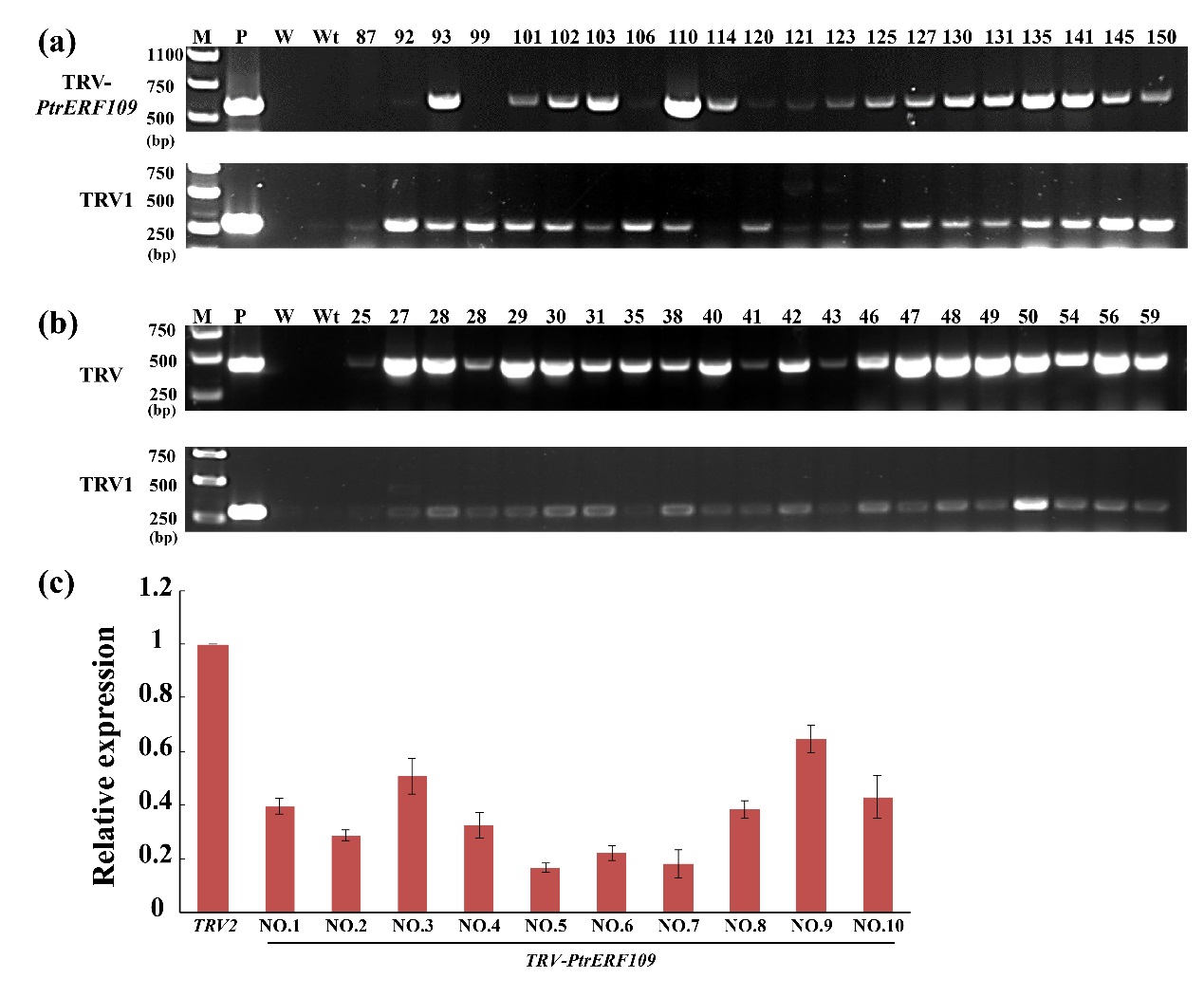


**Figure S5. Molecular characterization of the VIGS plants by genomic PCR and qPCR.** (a, b) Genomic PCR for identification of the TRV-*PtrERF109* (a) and TRV control (b) plants derived from the agro-infiltration. M, molecular marker; P, plasmid DNA (used as a positive control); W, ddH_2_O; Wt, wild type. (c) Expression of *PtrERF109* in ten positive VIGS lines, as analyzed by qPCR. *Actin* gene was used as an internal control. Error bars indicate ± SE (n = 4).


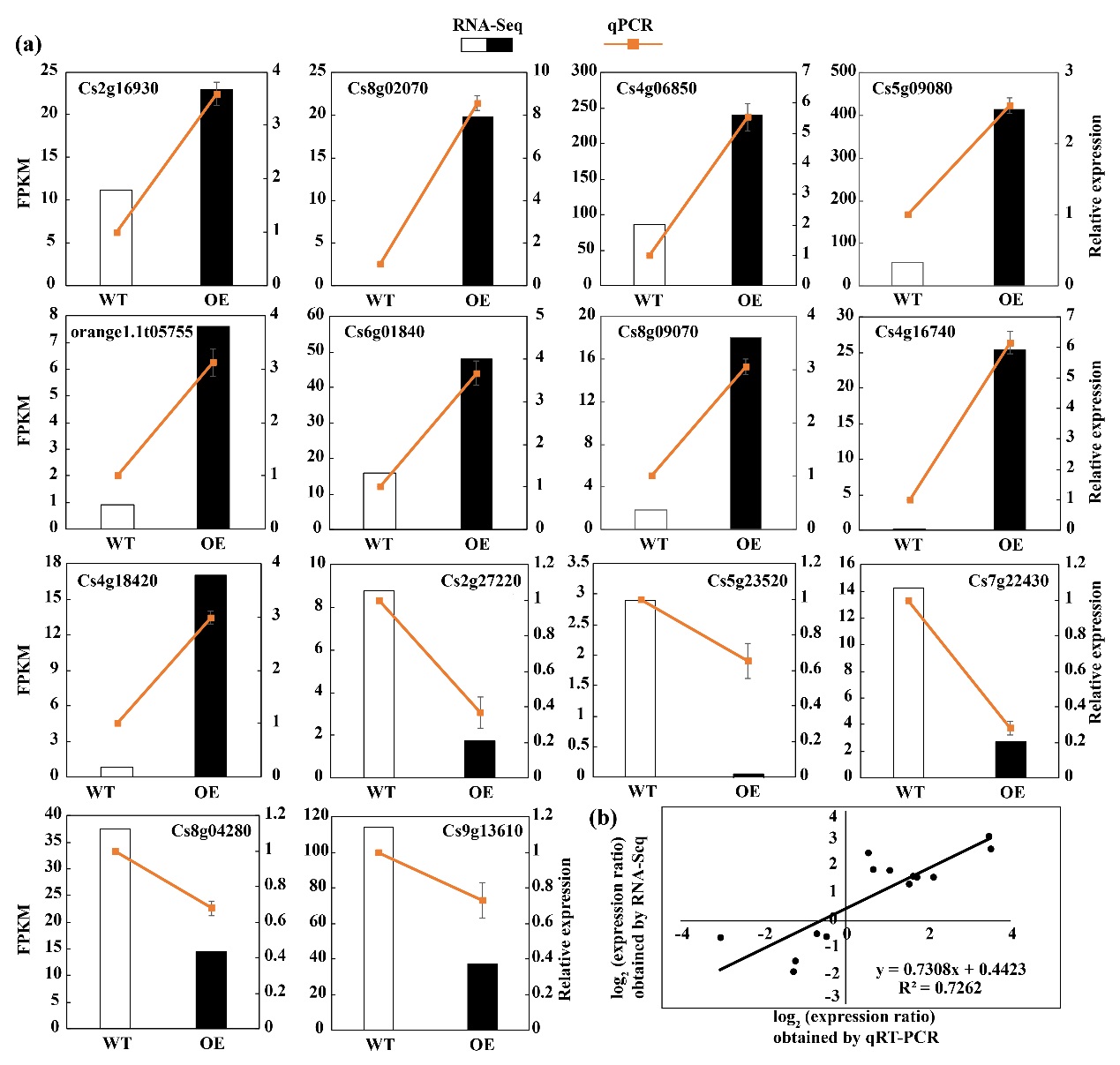


**Figure S6. Validation of differentially expressed genes by qPCR analysis.** (a) Transcript levels of 14 randomly selected DEGs, including 9 up-regulated and 5 down-regulated genes. The Y-axis on the left shows corresponding expression data of RNA-Seq (histogram), while Y-axis on the right shows the relative gene expression levels analyzed by qPCR (orange lines). The X-axis represents different samples, WT (wild type) and OE (overexpression line). *Actin* gene was used as an internal control. Error bars indicate ± SE (n = 4). (b) Comparison between the log_2_ of gene expression ratios obtained from RNA-Seq data and qPCR.
