# Supplementary material for: ERF109 of trifoliate orange (Poncirus trifoliata (L.) Raf.) contributes to cold tolerance by directly regulating expression of Prx1 involved in antioxidative process
Source: Plant Biotechnol J. 2019 Jan 4;17(7):1316–32. doi: 10.1111/pbi.13056 (PMC6576027; doi:10.1111/pbi.13056)
Supplement: Supplementary file 2 — Table S1 Summary of RNA‐sequencing data for the two replicates of each genotype. [file PBI-17-1316-s004.docx]

**Table S1.** Summary of sequencing data for each sample.

| **Sample** | **Sequencing Strategy** | **Raw Data Size (bp)** | **Raw Reads Number** | **Clean Data Size (bp)** | **Clean Reads Number** | | **Clean Data Rate (%)** |
| --- | --- | --- | --- | --- | --- | --- | --- |
| OE-1 | SE50 | 1206841900 | 24136838 | 1206626750 | | 24132535 | 99.98 |
| OE-2 | SE50 | 1206844200 | 24136884 | 1206544150 | | 24130883 | 99.97 |
| WT-1 | SE50 | 1206782800 | 24135656 | 1206586900 | | 24131738 | 99.98 |
| WT-2 | SE50 | 1206806350 | 24136127 | 1206547100 | | 24130942 | 99.97 |

Note: “OE” means *PtrERF109* overexpressing lemon, “WT” means wild type lemon.
