# Supplementary material for: ERF109 of trifoliate orange (Poncirus trifoliata (L.) Raf.) contributes to cold tolerance by directly regulating expression of Prx1 involved in antioxidative process
Source: Plant Biotechnol J. 2019 Jan 4;17(7):1316–32. doi: 10.1111/pbi.13056 (PMC6576027; doi:10.1111/pbi.13056)
Supplement: Supplementary file 5 — Table S4 List of primers used in this study. [file PBI-17-1316-s002.docx]

**Table S4.** List of primers used in this study.

| **Primer names** | **Purpose** | **Primer sequence** | |
| --- | --- | --- | --- |
|  |  | Forward primer | Reverse primer |
| GSP1 | Gene clone of *PtrERF10*9 | ATTCCAGAGCCAACACGAAC | GAACGTGGGATTTCGCCAGC |
| GSP2 | qRT-PCR analysis of *PtrERF109* | TGCAAAGATCCTCAAAGCGACC | CGTTGTCTGTGTTTCCGACGAG |
| GSP3 | Subcellular localization | CG*GGATCC*ATGCAAAGATCCTCAAAGCG | TCC*CCCGGG*TGAAGTAAGACCATTGGCAG |
| GSP4 | Transcriptional activation assay (Full-length of PtrERF109) | CATGGAGGCCGAATT*CCCGGG*ATGCAAAGATCCTCAAAGCG | CCGCTGCAGGTCGAC*GGATCC*TCATGAAGTAAGACCATTGGC |
| GSP5 | Transcriptional activation assay (PtrERF109ΔC) | CATGGAGGCCGAATT*CCCGGG*ATGCAAAGATCCTCAAAGCG | CCGCTGCAGGTCGAC*GGATCC*TGTCGTGCTATCGGGAAATGG |
| GSP6 | Transcriptional activation assay (PtrERF109ΔN) | CATGGAGGCCGAATT*CCCGGG*GTGGCAACAGCATACGAGCAG | CCGCTGCAGGTCGAC*GGATCC*TCATGAAGTAAGACCATTGGC |
| GSP7 | Overexpression vector construction | GC*TCTAGA*ATGCAAAGATCCTCAAAGCGAC | TCC*CCCGGG*TCATGAAGTAAGACCATTGGCAGTA |
| GSP8 | Transgenic plants identification *(35S*+Gene reverse primer) | TCCTCGGATTCCATTGCCCAGC | TGAAGTAAGACCATTGGCAG |
| GSP9 | NPT II | ATGACTGGGCACAACAGACAA | CGATACCGTAAAGCACGAGGA |
| GSP10 | Ubiquitin | GGTGTTTCCAGTGGCGGACG | TCCTCCCCTCAGCTACGGGGTAT |
| GSP11 | Actin | CATCCCTCAGCACCTTCC | CCAACCTTAGCACTTCTCC |
| GSP12 | qRT-PCR analysis of Cs6g01840 | AGATGGCAGATGAAGGCACAGC | ACTCTCTCACACACGCGCACAC |
| GSP13 | qRT-PCR analysis of Cs8g02070 | AAGGTTCTCAGTTCCACTGG | TCTCTGGGTCTAGGACAAAGC |
| GSP14 | qRT-PCR analysis of Cs4g16740 | AGAGAATAAGGAAGCGCAGGTG | AGGCTTTCCAGCAATGCCAG |
| GSP15 | qRT-PCR analysis of Cs5g09080 | AGGGTTCTACTAGCACCAACTG | AGGGTATCCCTAGGGCAAGTG |
| GSP16 | qRT-PCR analysis of Cs8g09070 | AGTACCCACCACTGCCCAAATG | ACTTTGGAATTGGAGGCAGTGG |
| GSP17 | qRT-PCR analysis of Cs4g06850 | TGCTTGTAGCCGAGTACCTGCAC | AGCTTCGCAGAAAGGTGGCGGT |
| GSP18 | qRT-PCR analysis of Cs4g18420 | AGCACATTTCACAGCAAGACCTC | AGTGACATCTTGACCAGCAAGAC |
| GSP19 | qRT-PCR analysis of orange1.1t05755 | AGGTACAACAACCGTGGAAGG | ACACCTTTAGTTCCAGCAGG |
| GSP20 | qRT-PCR analysis of Cs2g16840 | TCATGAGGGTGACTGGGAGAG | TCCAAAGAGTGAAGGTAGCTTGG |
| GSP21 | qRT-PCR analysis of Cs2g16930 | TGTCTCTAGCTGGTGAGTTGG | TCACAACTTTGAACGCTCTGAGG |
| GSP22 | qRT-PCR analysis of *Prx1* | AGAGCTGTTTTCGACTCCCG | CATTGACCCTCCTGCAGTTC |
| GSP23 | pGADT7-*PtrERF109* forY1H | GACGTACCAGATTACGCT*CATATG*ATGCAAAGATCCTCAAAGCG | TCGATGCCCACCCGGGTG*GAATTC*TCATGAAGTAAGACCATTGGCAG |
| GSP24 | pAbAi-*Prx1* forY1H | CTTGAATTCGAGCTC*GGTACC*CCGAAGGGTTTCGATTTGTGG | ATACAGAGCACATGC*CTCGAG*CGTGAACTTATTTGGTGGCTGC |
| GSP25 | pGreen II 62-SK-*PtrERF109* for LUC | ATCGATACCGTCGAC*CTCGAG*ATGCAAAGATCCTCAAAGCG | TGATTTCAGCGAATT*GGTACC*TCATGAAGTAAGACCATTGGCAG |
| GSP27 | pGreen II 0800-LUC-*Prx1* for LUC | CTTGAATTCGAGCTC*GGTACC*CCGAAGGGTTTCGATTTGTGG | ATACAGAGCACATGC*CTCGAG*TTGAAGAGGTTAGTGAGAGTG |
| GSP28 | pDONR207-*PtrERF109* as entry clone vector for constructing protein expression vector | GGGGACAAGTTTGTACAAAAAAGCAGGCTCCATGCAAAGATCCTCAAAGCG | GGGGACCACTTTGTACAAGAAAGCTGGGTCTGAAGTAAGACCATTGGCAG |
| GSP29 | TRV-*PtrERF109* for VIGS | AGAAGGCCTCCATGG*GGATCC*GAATTCGGCGGCGGCAACAG | TGTCTTCGGGACATG*CCCGGG*CGATCTCCAAATTGATGGTC |
| GSP30 | PCR identification of TRV-*PtrERF109* in VIGS plants | ATTCACTGGGAGATGATACGCT | CGATCTCCAAATTGATGGTC |
| GSP31 | PCR identification of TRV1 vector in VIGS plants | ATTGAGGCGAAGTACGATGG | CCATCCACAATTATTTTCCGC |
| GSP32 | PCR identification of pTRV2 empty vector in VIGS plants | ATTCACTGGGAGATGATACGCT | AGTCGGCCAAACGCCGATCTCA |
| GSP33 | pPtrERF109:GUS | CTACAGCGCTAAGCTTGGCTGCAGCGCCGATTTGAACAAAGTCTTTAC | AAGGGACTGACCACCCGGGGATCCTTCGTTGTCTGTGTTTCCGACG |
