# Supplementary material for: ERF109 of trifoliate orange (Poncirus trifoliata (L.) Raf.) contributes to cold tolerance by directly regulating expression of Prx1 involved in antioxidative process
Source: Plant Biotechnol J. 2019 Jan 4;17(7):1316–32. doi: 10.1111/pbi.13056 (PMC6576027; doi:10.1111/pbi.13056)
Supplement: Supplementary file 6 — Table S5 Accession numbers of the ERF genes used for phylogenetic tree construction. [file PBI-17-1316-s006.docx]

**Table S5.** Accession numbers of ERF genes used for phylogenetic tree construction.

| **Gene name** | **Gene ID** | **Species** |
| --- | --- | --- |
| LeERF2 | NP_001234308.2 | *Lycopersicon esculentum* |
| LeERF5 | AAS72389.1 | *Lycopersicon esculentum* |
| TaERF3 | ABQ52687.1 | *Triticum aestivum* |
| TaPIE1 | EF583940.1 | *Triticum aestivum* |
| OsEREBP1 | Os02g0782700 | *Oryza sativa* L. |
| OsERF109 | XP_015649367.1 | *Oryza sativa* L. |
| AtERF1 | AT3G23240 | *Arabidopsis thaliana* |
| AtERF2 | AT5G47220 | *Arabidopsis thaliana* |
| AtERF3 | AT1G50640 | *Arabidopsis thaliana* |
| AtERF4 | AT3G15210 | *Arabidopsis thaliana* |
| AtERF6 | AT4G17490 | *Arabidopsis thaliana* |
| AtERF7 | AT3G20310 | *Arabidopsis thaliana* |
| AtERF73 | AT1G72360 | *Arabidopsis thaliana* |
| AtERF74 | AT1G53910 | *Arabidopsis thaliana* |
| AtERF96 | AT5G43410 | *Arabidopsis thaliana* |
| AtERF98 | AT3G23230 | *Arabidopsis thaliana* |
| AtERF105 | AT5G51190 | *Arabidopsis thaliana* |
| AtERF109 | AT4G34410 | *Arabidopsis thaliana* |
| AtRAP2.2 | AT3G14230 | *Arabidopsis thaliana* |
| AtRAP2.6 | AT1G43160 | *Arabidopsis thaliana* |
| ABR1 | AT5G64750 | *Arabidopsis thaliana* |
| CRF1 | AT4G11140 | *Arabidopsis thaliana* |
| CRF5 | AT2G46310 | *Arabidopsis thaliana* |
| SHINE2 | AT5G11190 | *Arabidopsis thaliana* |
| SHINE3 | AT5G25390 | *Arabidopsis thaliana* |
| GmERF3 | ACD47129.1 | *Glycine max* |
| GmERF4 | ACE76905.1 | *Glycine max* |
| SodERF3 | CAM35490.1 | *Saccharum officinarum* |
| CaERFLP1 | AAS20427.1 | *Capsicum annuum* |
| CaAIEF1 | ARR75181.1 | *Capsicum annuum* |
| MsERF8 | AEQ64868.1 | *Medicago sativa* |
